# Multi-modal profiling of human fetal liver hematopoietic stem cells reveals the molecular signature of engraftment

**DOI:** 10.1038/s41467-022-28616-x

**Published:** 2022-03-01

**Authors:** Kim Vanuytsel, Carlos Villacorta-Martin, Jonathan Lindstrom-Vautrin, Zhe Wang, Wilfredo F. Garcia-Beltran, Vladimir Vrbanac, Dylan Parsons, Evan C. Lam, Taylor M. Matte, Todd W. Dowrey, Sara S. Kumar, Mengze Li, Feiya Wang, Anthony K. Yeung, Gustavo Mostoslavsky, Ruben Dries, Joshua D. Campbell, Anna C. Belkina, Alejandro B. Balazs, George J. Murphy

**Affiliations:** 1 Section of Hematology and Medical Oncology, School of Medicine, Boston University, Boston, MA USA; 2 Center for Regenerative Medicine (CReM), Boston University and Boston Medical Center, Boston, MA USA; 3 Division of Computational Biomedicine, School of Medicine, Boston University, Boston, MA USA; 4 Ragon Institute of MGH, MIT and Harvard, Cambridge, MA USA; 5 Department of Pathology and Laboratory Medicine, School of Medicine, Boston University, Boston, MA USA; 6 Flow Cytometry Core Facility, School of Medicine, Boston University, Boston, MA USA

**Keywords:** Haematopoietic stem cells, Haematopoietic stem cells, Multipotent stem cells, Haematopoiesis

## Abstract

The human hematopoietic stem cell harbors remarkable regenerative potential that can be harnessed therapeutically. During early development, hematopoietic stem cells in the fetal liver undergo active expansion while simultaneously retaining robust engraftment capacity, yet the underlying molecular program responsible for their efficient engraftment remains unclear. Here, we profile 26,407 fetal liver cells at both the transcriptional and protein level including ~7,000 highly enriched and functional fetal liver hematopoietic stem cells to establish a detailed molecular signature of engraftment potential. Integration of transcript and linked cell surface marker expression reveals a generalizable signature defining functional fetal liver hematopoietic stem cells and allows for the stratification of enrichment strategies with high translational potential. More precisely, our integrated analysis identifies CD201 (endothelial protein C receptor (EPCR), encoded by *PROCR*) as a marker that can specifically enrich for engraftment potential. This comprehensive, multi-modal profiling of engraftment capacity connects a critical biological function at a key developmental timepoint with its underlying molecular drivers. As such, it serves as a useful resource for the field and forms the basis for further biological exploration of strategies to retain the engraftment potential of hematopoietic stem cells ex vivo or induce this potential during in vitro hematopoietic stem cell generation.

## Abstract (web summary)

During human embryonic development, haematopoietic stem cells of the foetal liver undergo expansion, while retaining engraftment capacity. Here the authors apply CITE-seq to profile single cells from a human foetal liver, identifying a molecular signature of engraftment potential

## Introduction

The human hematopoietic stem cell (HSC) has been the focus of intense study due to its remarkable regenerative potential and its therapeutic utility in treating a variety of diseases. HSCs reside at the apex of the hematopoietic system and have the capacity to both self-renew and differentiate into all mature blood cell types. As such, these cells, and the hierarchical structure of their progeny, represent an ideal system in which to ask fundamental biological questions and discover key insights into hematopoietic development and disease. These insights could be harnessed to improve ex vivo expansion methods, increase engraftment efficiency, and enable in vitro HSC generation from pluripotent stem cell (PSC) sources.

The first HSCs emerge in the aorta-gonad-mesonephros (AGM) region prior to traveling to the fetal liver (FL), where they undergo expansion to create the HSC pool that sustains hematopoiesis for the lifetime of an individual. The FL remains the main site of hematopoiesis until the bone marrow (BM) becomes competent to serve as the final HSC niche closer to birth^1–4^. This dynamic transition is coupled to a switch from a proliferative to a predominantly quiescent phenotype postnatally^2^, reflecting just one of several differences between developmentally distinct HSCs^5^. A key functional difference between FL-derived and more mature HSCs is the superior engraftment potential of FL when compared to cord blood (CB) and BM cells^6^. The retention of robust engraftment potential during active expansion highlights a unique feature of FL HSCs, as proliferation and HSC functionality are inversely correlated postnatally^7,8^. This prompted us to specifically profile FL-derived HSCs to establish a detailed molecular signature of engraftment potential.

To dissect the molecular underpinnings of engraftment potential at the highest possible resolution, we combined several orthogonal single-cell profiling approaches. The emergence of single-cell RNA sequencing (scRNAseq) technologies has led to significant advances in terms of characterization of the larger hematopoietic stem and progenitor cell (HSPC) pool. Transcriptomic profiling of large numbers of single CD34^+^ cells has resulted in a more nuanced understanding of the postnatal HSPC compartment, revealing a continuum of transcriptional states rather than a succession of clearly demarcated progenitor stages^9–12^. Recent work has extended such large-scale single-cell transcriptomic profiling efforts to the prenatal stage^13^, including work in the context of the developmental cell atlas analyzing the dynamic hematopoietic composition of the entire FL and other tissues throughout early human development^14^. However, despite interrogation of large numbers of total cells, their relative scarcity resulted in the profiling of few truly functional HSCs.

In this study, we specifically focus on this rare HSC fraction by profiling a highly enriched population of FL HSCs that have been confirmed to be functional for engraftment. Several strategies exist to enrich for functional HSCs within a pool of HSPCs^15–18^, with a combination of GPI-80 and CD133 resulting in one of the highest frequencies described (~1/5)^16^. Glycophosphatidylinositol-anchored surface protein GPI-80 has been shown to mark a subpopulation of FL HSCs that combine self-renewal ability and engraftment potential^17^. Using this marker as the basis for functional HSC enrichment as confirmed in transplantation assays, we single-cell profile these highly enriched FL cells to uncover the detailed molecular signature of engraftable FL HSCs. Further dissection of this engraftment profile reveals signatures highlighting the importance of proteome integrity maintenance, as well as the prominent expression of factors linked to aging and the concomitant decline of HSC functionality. This comprehensive characterization of the engraftment signature of FL HSCs using multi-modal profiling to define an essential biological function at a key developmental timepoint will serve as a useful resource for the field. Our interactive dataset is freely available to the scientific community through the following platform: https://engraftable-hsc.cells.ucsc.edu

## Results

### The hematopoietic landscape of the human fetal liver

To obtain a detailed molecular signature of human FL HSCs, we performed CITE-seq^19^, a technique that allows for the simultaneous assessment of transcript and cell-surface marker level expression by combining droplet based single-cell RNA sequencing (scRNAseq) and oligo-tagged antibodies (Supplementary Table 1). Following dissociation of a human FL sample, cells were divided into either CD34^+^-enriched or CD34^−^ flowthrough cells via magnetic bead separation (Supplementary Fig. 1a). The CD34^−^ live cells were further subdivided into GYPA^+^ and GYPA^−^ via fluorescence activated cell sorting (FACS) to capture populations of maturing erythroid progenitors that constitute a sizeable portion of the FL at this stage in development (CD34^−^GYPA^+^). From the CD34^+^-enriched compartment, live CD34^+^ cells were sorted (CD34^+^bulk) to reflect the cell population that is routinely used in a clinical HSC transplantation setting. In a separate fraction, we further enriched this population using GPI-80 expression (GPI-80^+^), a marker tightly linked to engraftment potential^16,17^, to focus our analysis on HSCs capable of long-term engraftment. Following data processing and quality control, this resulted in a total of 26,407 FL cells profiled from one fetal liver, divided across the following fractions: 8735 CD34^+^bulk cells, 7235 GPI-80^+^ cells, 6793 CD34^−^GYPA^−^ cells and 3644 CD34^−^GYPA^−^ cells (Supplementary Fig. 1a).

**Fig. 1:**
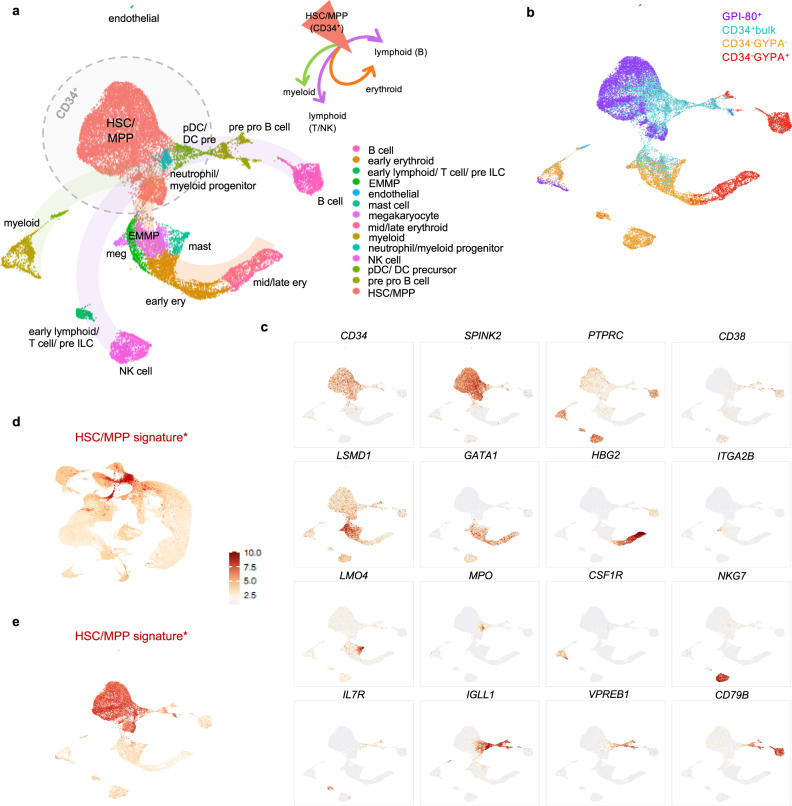
The hematopoietic landscape of human fetal liver at single-cell resolution. **a** UMAP representation of the transcriptomic analysis of all four FL fractions combined showing the cell types identified in each cluster. Cells are colored by cell lineage annotation, developed using the expression of key markers and DEG from the transcriptomic analysis. Innate lymphoid cell precursor (pre ILC), erythroid-megakaryocyte-mast progenitor (EMMP), natural killer (NK) cell, plasmacytoid dendritic cell (pDC), dendritic cell precursor (DC precursor), hematopoietic stem cell/multipotent progenitor (HSC/MPP). This combined analysis represents different fractions isolated from one FL sample (Fig. S1). **b** UMAP representing the distribution of each FL fraction within the combined analysis. Cells are colored by the fraction of origin of each cell. **c** UMAP overlays illustrating mRNA expression patterns of a set of HSC/MPP and lineage markers. The scale bar applies to the scaled expression shown in panels **c**–**e**. **d** Re-analysis of data from Popescu et al. to illustrate the identified HSC/MPP signature in its original context^14^. **e** Overlay of HSC/MPP signature identified in Popescu et al. onto the combined transcriptomic analysis described in this study.

Combinatorial transcriptomic analysis of all four fractions resulted in an overview of the hematopoietic landscape at this developmental stage (Fig. 1a). Figure 1b displays the distribution of the four fractions within the combined dataset, illustrating that the CD34^+^ HSC/multipotent progenitor (MPP) compartment represents cells belonging to the CD34^+^ bulk and GPI-80^+^ enriched fractions. Greater than half of all assayed cells represented CD34^+^ HSCs/MPPs (Fig. 1a–e), which is further emphasized by the overlay of a previously established HSC/MPP signature^14^ within the human FL (Fig. 1d) onto our combined data (Fig. 1e). Taking these CD34^+^ HSCs/MPPs as a starting point, gene expression changes over pseudotime were assessed for each major hematopoietic lineage. This analysis confirmed downregulation of HSC/MPP marker genes and upregulation of key lineage identity genes as commitment progresses, reflecting the expected hematopoietic cell types at this developmental stage (Fig. 1e and Supplementary Fig. 1b–e).

### Transcriptomic profiling of engraftment potential

**Fig. 2:**
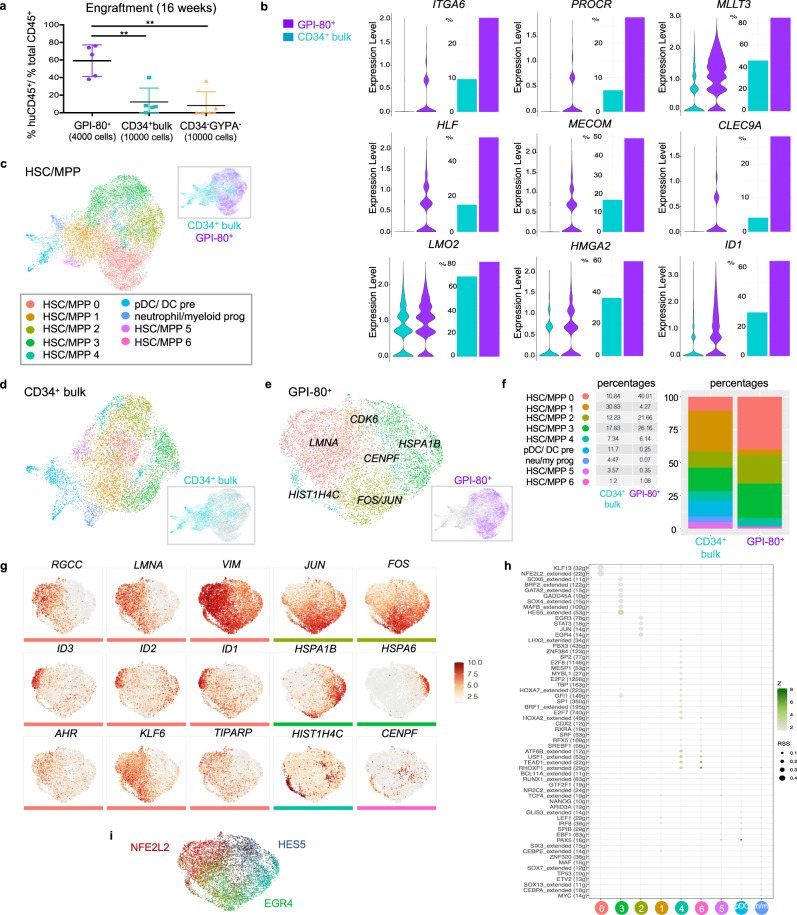
Defining the molecular signature of FL-derived HSCs with the highest engraftment potential. **a** Engraftment data demonstrating superior engraftment of the GPI-80^+^ fraction 16 weeks post transplantation. Five NSG mice were transplanted per condition with 10,000 cells each (4000 cells for the GPI-80+ condition) and engraftment was assessed by determining the ratio (%) of peripheral blood cells expressing human CD45 versus total CD45 expressing cells. The mean is shown with error bars representing the standard error of the mean. One-way analysis of variance (ANOVA) was performed with Tukey test to account for multiple comparisons and obtain adjusted *p*-values: GPI-80^+^ vs. CD34^+^bulk *p* = 0.002, GPI-80^+^ vs. CD34^-^GYPA^-^ *p* = 0.001 (**p < 0.01). Source data are provided as a Source Data file. **b** Violin plots of differentially expressed genes between HSCs/MPPs in the GPI-80^+^ and CD34^+^ bulk fractions (left) and bar plots illustrating the percentage of cells expressing these genes per fraction (right). **c** UMAP representation showing the clusters identified in the CD34^+^ HSC/MPP population. **d** UMAP representation of the HSCs/MPPs corresponding to the CD34^+^ bulk sample, retaining the cluster definitions established in panel **c**. **e** UMAP representation of the HSCs/MPPs corresponding to the GPI-80^+^ sample, retaining the cluster definitions established in panel **c**. Additional labeling with one of the top most enriched genes is shown for each cluster (see Table S4). **f** Cluster dynamics upon GPI-80-enrichment showing the proportion of each cluster before (CD34^+^bulk) and after (GPI-80^+^) functional enrichment in a stacked bar plot. Percentages of cells in each cluster are shown on the left. **g** UMAP overlays illustrating mRNA expression patterns of genes enriched in different GPI-80^+^ clusters. The scale bar shows scaled expression from 0-Max. **h** Regulon specificity score (RSS) plot showing the regulons with the highest specificity for a given cluster within the GPI-80^+^ fraction. The scale bar represents *z*-scores and dot sizes reflect the RSS for each regulon. **i** Overlay of the expression patterns of cluster-specific regulons onto the GPI-80^+^ UMAP.

In parallel with capture for CITE-seq, cells from these same sorted fractions (with the exception of CD34^−^GYPA^+^) were used in simultaneous transplantation experiments to assess engraftment capacity. These experiments revealed superior per-cell engraftment potential of the GPI-80^+^ fraction as compared to CD34^+^bulk and CD34^−^GYPA^−^ fractions, confirming enrichment for bona fide functional HSCs in this population (Fig. 2a). Although the GPI-80^+^ fraction represented only 2.37% of the cells within the CD34^+^bulk population (Supplementary Fig. 1a), sorting and enrichment of this fraction enabled the analysis of 7235 GPI-80^+^ cells, resulting in profiling of the engraftable FL HSC at unprecedented resolution.

In concordance with their superior engraftment potential, we found enrichment for known HSC markers such as *ITGA6* (CD49f), *PROCR* (CD201 or EPCR), *MLLT3*, *HLF*, *MECOM*, *CLEC9A*, *LMO2*, and *HMGA2* in the GPI-80^+^ fraction compared to CD34^+^bulk cells (Fig. 2b). Enrichment for these genes, and others with currently unexplored connections to HSCs, distinguishes the GPI-80^+^ fraction from bulk CD34^+^ cells, revealing a detailed transcriptomic signature that marks engraftable FL HSCs. A comprehensive list of all differentially expressed genes (DEGs) that make up this engraftment signature is presented in Supplementary Table S2.

To further analyze engraftable FL HSCs, we first focused on the CD34^+^ HSC/MPP population (Fig. 1c) captured within the gray dotted circle in Fig. 1a. Separate clustering of this subset resulted in seven distinct HSC/MPP clusters (HSC/MPP 0–6) in addition to previously identified progenitor clusters (Fig. 2c). To determine which of these clusters best correlated with engraftment potential, we tracked cluster dynamics upon GPI-80^+^ enrichment. Using the cluster designations from Fig. 2c, we identified corresponding clusters in the individual fractions making up this CD34^+^ HSC/MPP population (Fig. 2d, e). Mapping the changes in cluster proportions between CD34^+^ bulk and GPI-80^+^ enriched fractions (Fig. 2f), revealed a relative absence of cells corresponding to HSC/MPP cluster 1 within the GPI-80+ enriched fraction, suggesting that these cells and their associated transcriptomic signature likely contribute minimally to the engraftment potential of FL HSCs. Emboldening this point, one of the top enriched genes in this cluster is the activation marker *CDK6*, whose expression marks the transition towards more differentiated progenitors^20^. Similarly, we noted a significant reduction in cluster 5 and those clusters corresponding to pDC/DCpre cells and neutrophil/myeloid progenitors. Conversely, the proportion of cells in clusters 0, 2, and 3 increased in the GPI-80^+^ fraction. Of these, the most pronounced change was noted for HSC/MPP cluster 0, which showed a substantial fourfold increase. This specific enrichment, resulting in cluster 0 cells accounting for 40% of the total GPI-80^+^ population, strongly suggests that the transcriptomic profile corresponding to this cluster best represents cells with functional engraftment potential.

To further dissect the gene expression signature specific to cluster 0, we assessed the differential gene expression between this and other GPI-80^+^ clusters. *RGCC* and *LMNA* were identified as the top enriched genes in cluster 0, closely followed by *VIM* and *ID1* and *ID3* (Fig. 2g, Supplementary Table 3, and Supplementary Fig. 2a). In addition to ID signaling pathway members, whose expression appears concentrated along the outer edge of cluster 0, we also found enrichment for *KLF6, AHR* and *TIPARP*, encoding key players in the aryl hydrocarbon receptor (AHR) pathway where the latter two make up an AHR-TIPARP-negative feedback axis^21^ (Fig. 2g and Supplementary Table 3). Interestingly, *LMNA* was found to be more prominently expressed in FL than postnatal CD34^+^ and HSC-enriched fractions (Supplementary Fig. 2b) in line with its previously described inverse correlation with ageing^22^. Similarly, the expression of several other cluster 0 genes such as *ID1*, *ID3*, *AHR*, *TIPARP*, and *HES1* was more pronounced in FL compared to postnatal HSCs, suggesting the downregulation of these genes over developmental time (Supplementary Fig. 2c). Moreover, gene regulatory network (GRN) analysis using Single-cell Regulatory Network Inference and Clustering (SCENIC)^23^ revealed that NFE2L2, KLF13 and KLF10 regulons drive a cluster-0-specific transcriptional program (Fig. 2h, i and Supplementary Fig. 2d, e). Notably, KLF13 and KLF10 both regulate *LMNA* expression in addition to KLF10 regulating itself as well as *KLF13* (Supplementary Fig. 2e). NFE2L2 is a negative regulator of cell cycle entry in mouse HSCs where it actively maintains a balance between quiescence and self-renewal^24^. In line with this role, we find that the majority of cells belonging to cluster 0 are in a quiescent state (Supplementary Fig. 3a).

Although less prominent than what we observed for cluster 0, the proportion of cells in clusters 2 and 3 also increased upon GPI-80 enrichment. Of these, cluster 2 was characterized by enrichment for *FOS* and *JUN*, two factors involved in numerous signaling pathways (Fig. 2g, Supplementary Table 3, and Supplementary Fig. 3b). GRN analysis for this cluster further emphasized involvement of the immediate early response transcription factor family with regulons such as JUN, EGR4, EGR3 and EGR1 characterizing this particular cluster (Supplementary Fig. 3c). Interestingly, cluster 3 was enriched for heat shock proteins (*HSPA1A*, *HSPB1B*, *HSPA6*, *HSPB1*), suggesting that the unfolded protein response best distinguishes this population (Supplementary Fig. 3d). In contrast to cluster 0 where HES1 was identified as one of the top most active regulons (Supplementary Fig. 2d), HES5 regulates a cluster 3-specific transcriptional program (Supplementary Fig. 3e). Like cluster 0, clusters 2 and 3 contain a majority of cells in G1(G0) and enrichment of these 3 clusters upon GPI-80 selection resulted in an increase in quiescence compared to the CD34^+^bulk fraction at the expense of actively cycling cells (Supplementary Fig. 3a).

### Linking cell-surface protein expression to the transcriptome

**Fig. 3:**
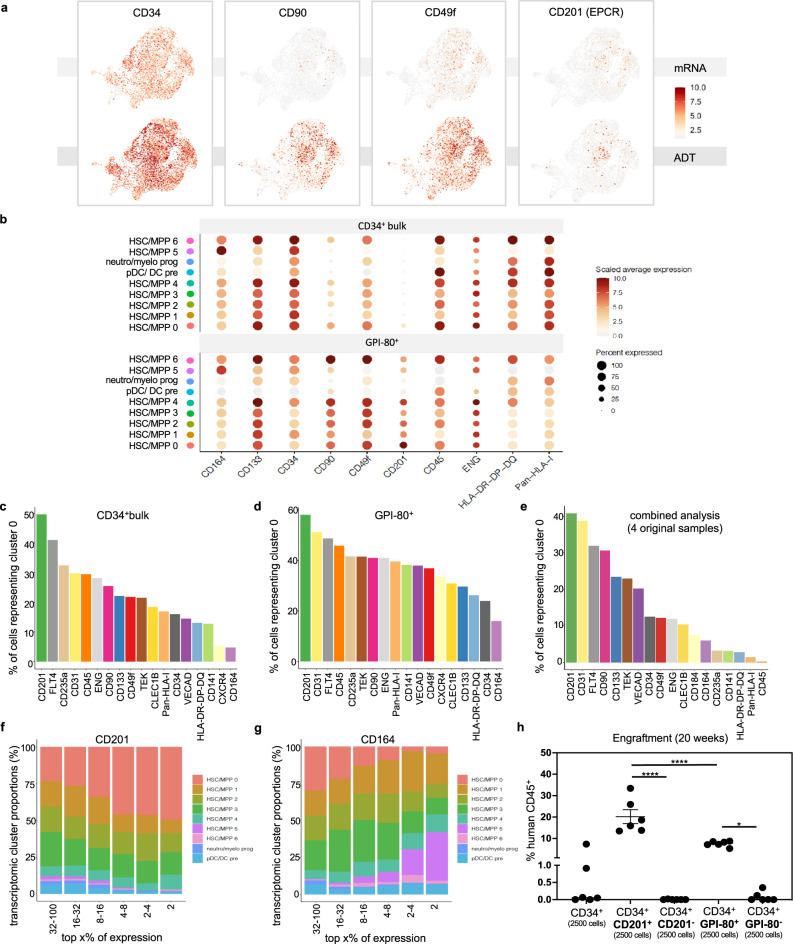
Integration of mRNA and antibody-derived tag (ADT) data identifies CD201 as a marker enriching for highly functional HSCs. **a** Comparison of mRNA and ADT expression for a selection of HSC markers in the CD34^+^bulk sample. **b** Dot plot illustrating the distribution of antibody-derived tag (ADT) expression for a selection of HSC markers in the GPI-80^+^ fraction versus the CD34^+^ bulk fraction. Expression is represented as scaled average expression and the scale is kept constant between the CD34^+^ bulk and GPI-80^+^ fraction for each marker to allow for comparison of relative intensities. “Percent expressed” represents the fraction of cells having non-zero expression values for each cell cluster in the two samples. **c**–**e** Comparison of the ability to enrich for cluster 0 across the different cell-surface markers included in our ADT panel for the CD34^+^bulk fraction (**c**), GPI-80^+^ fraction (**d**), and combined analysis of all 4 originally sorted samples (**e**). The proportion of cells representing cluster 0 (%) is shown on the *y*-axis, considering cells enriched for each marker (top 5%) on the *x*-axis. **f**, **g** Transcriptomic cluster composition (%) within the CD34^+^ bulk fraction upon stepwise enrichment for cell-surface marker expression of CD201 (**f**) and CD164 (**g**). The *x*-axis is divided in segments showing the indicated top x% of expression for each marker. **h** Engraftment data demonstrating superior engraftment of the CD34^+^CD201^+^ fraction compared to the CD34^+^GPI-80^+^ fraction 20 weeks post transplantation. Six NSG mice were transplanted per condition with 2500 cells each and engraftment was assessed by determining the percentage (%) of peripheral blood cells expressing human CD45. The mean is shown for each condition with error bars representing the standard error of the mean. One-way analysis of variance (ANOVA) was performed with Tukey test to account for multiple comparisons and obtain adjusted *p*-values: *p = 0.0179, ****p < 0.0001. Source data are provided as a Source Data file. Longitudinal analysis and demonstration of multilineage repopulation can be found in Supplementary Fig. 4f–h.

Sequencing of antibody-derived tags (ADTs) enabled the coupling of transcriptional data with cell-surface marker expression. Overall, a strong correlation was found between mRNA and ADT expression, with the latter providing increased coverage and an additional layer of information^19^ (Fig. 3a and Supplementary Fig. 4a, b). Figure 3a projects both layers of information onto the CD34^+^bulk UMAP for a selection of HSC markers. Notably, for CD34, CD90 and CD49f, often used in combination to enrich for HSCs, ADT expression patterns were observed that were not readily apparent based on mRNA data alone, indicating co-expression of these markers at the protein level. Importantly, ADT data also highlighted CD201 as a marker for cluster 0 and our engraftment signature (Fig. 3a, b and Supplementary Fig. 4b). CD201, also known as endothelial protein C receptor (EPCR) and encoded by *PROCR*, marks LT-HSCs in both mice and humans and appears to be stably retained ex vivo^7,18,25,26^. While not differentially expressed at the transcriptional level (Supplementary Table 3), enrichment for cluster 0 by CD201 surface expression was evident for both CD34^+^ HSC/MPP fractions (Fig. 3b–d) and also throughout the 26,407 combined cells from all 4 original fractions, irrespective of CD34 enrichment (Fig. 3e), suggesting that CD201 could be harnessed as a single marker to specifically enrich for highly functional FL HSCs. To this point, progressive in silico enrichment for CD201 expression demonstrated a robust increase in the proportion of cells corresponding to the engraftment signature represented by cluster 0 (Fig. 3f). Notably, 49% of the top CD201 expressing cells within the CD34^+^ bulk fraction represented cluster 0 cells versus ~25% when considering markers such as CD49f, CD90 and CD133 (Fig. 3c–f). This level of enrichment for cluster 0 even surpassed that observed when using GPI-80 to sort functional HSCs from bulk CD34^+^ cells (Fig. 2f). In stark contrast to CD201, enrichment based on surface expression of CD164, a marker described as an alternative to CD38^−^ gating in HSC enrichment strategies^11^, was correlated with a decrease in cluster 0 frequency (Fig. 3c, g). Progressive enrichment for HSC markers such as CD90, CD49f and ENG (endoglin, CD105) did not alter the transcriptomic profile of cells (Supplementary Fig. 4c–e).

To functionally validate the engraftment potential of cluster 0 cells and their enrichment based on CD201 surface expression, we transplanted CD201^+^ and CD201 depleted cells into conditioned NSG (NOD/SCID/*IL2rγ*^*null*^) mice and compared their engraftment capacity to GPI-80^+^ and GPI-80 depleted cells. These assays highlighted a robust enrichment in engraftable HSCs in both the GPI-80^+^ and CD201^+^ fractions as compared to the depleted fractions with superior engraftment of the CD201^+^ cells as compared to the GPI-80^+^ fraction (*p* $<$ 0.0001) (Fig. 3h). Both CD201 and GPI-80 enriched fractions showed robust multilineage reconstitution when followed for up to 20 weeks post transplantation (Supplementary Fig. 4f, g). Altogether, these findings illustrate that cell-surface protein expression coupled with transcript-level data provides a powerful approach towards classifying potential enrichment strategies for highly functional HSCs. It also provides a complimentary, yet orthogonal methodology with which to stratify the heterogeneity that exists within the HSC/MPP compartment of the FL.

### Multi-dimensional flow cytometry characterization

**Fig. 4:**
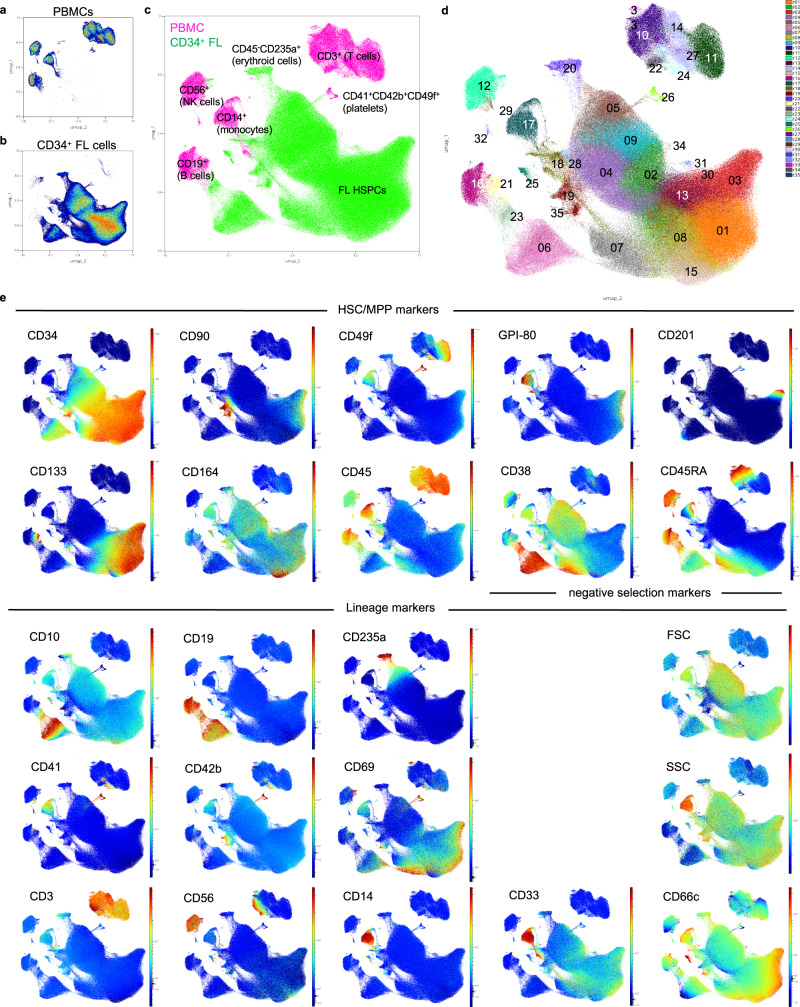
Multi-dimensional flow cytometric characterization across multiple FL samples validates antibody-derived tag (ADT) expression patterns. **a**, **b** Individual representation of peripheral blood mononuclear cells (PBMCs) (**a**) and CD34^+^ FL cells (**b**) in the same UMAP space. Color indicates plot density. **c** UMAP representation of both PBMCs (pink) and CD34^+^FL cells (green). Major mature blood cell subsets are indicated on the PBMC sample for orientation purposes. **d** UMAP representing the different Phenograph clusters identified in the combined dataset (PBMCs and CD34^+^ FL cells). **e** Expression patterns of individual markers and light scatter cytometric parameters overlaid onto the UMAP representation of the combined data. Color indicates fluorescence or light scatter signal intensity.

To further extend the cell-surface marker characterization of FL HSPCs, five additional CD34^+^ FL samples (post conception weeks 16-22) were profiled by flow cytometry using a 21-marker antibody panel (Supplementary Table 5). To enable comprehensive analysis of the resulting multi-dimensional dataset, marker expression was projected onto a common UMAP scaffold analogous to representations of high-dimensional transcriptomic data. Adult peripheral blood mononuclear cells (PBMCs) were simultaneously phenotyped and projected into the same UMAP space to provide context for less mature FL cell subsets. Phenograph clustering^27^ was performed on the combined data (PBMCs and FL CD34^+^ cells) (Fig. 4a–d and Supplementary Fig. 5a, b) and each of the identified FL clusters was populated with cells from more than one FL sample (Supplementary Fig. 5a, c). In addition to HSC markers present in our CITE-seq panel, lineage markers were also included to identify committed progenitors within the CD34^+^ FL fraction. UMAP visualization of the combined flow data from 5 CD34^+^ FL samples allowed for assessment of the overlap between CD34 expression and that of negative selection markers such as CD38 and CD45RA, as well as more committed lineage markers (Fig. 4e). This process allowed for clear sub-fractionation of the mature PBMCs (Fig. 4d, e) and demonstrated that certain mature cell markers (CD66c, CD33) are also broadly expressed within the CD34^+^ population, highlighting the importance of carefully choosing such markers in negative selection strategies^28^. Notably, when looking at the portion of the UMAP that represents CD34^+^CD38^−^CD45RA^−^ cells (clusters 1, 3, 8, 13, 30, 31 in Fig. 4d), cells that also co-express CD90 and CD49f (right edge of clusters 1 and 3) were identified as suggested by our ADT data (Fig. 3a). Interestingly, this visualization drew attention to a population of cells at the tip of cluster 3 in which CD49f, CD201 and GPI-80 appeared to be co-expressed (Fig. 4d, e). Protein-level co-expression of these HSC markers could be inferred based on comparison of ADT expression patterns in the CD34^+^bulk and GPI-80^+^ fractions as well (Fig. 3a, b and Supplementary Fig. 4b). Taken together, this multi-dimensional flow characterization confirms the expression patterns observed with ADTs and validates these findings across biologically distinct FL samples.

### Multi-modal comparison of HSC enrichment strategies

**Fig. 5:**
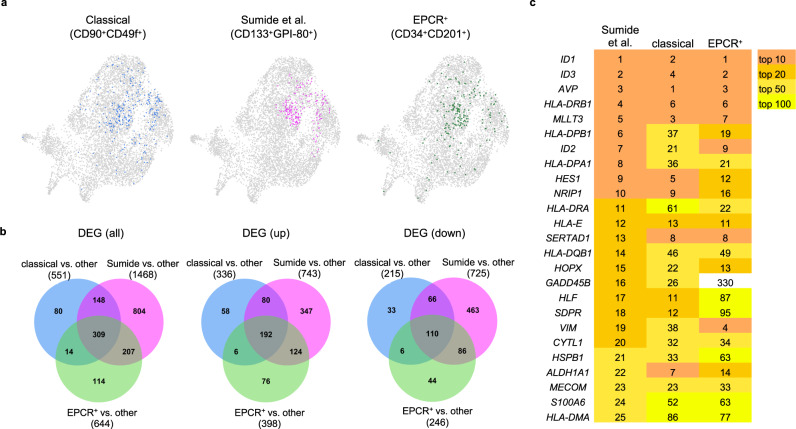
In silico comparison of HSC enrichment strategies reveals considerable transcriptomic overlap between signatures. **a** Overlays of in silico sorted HSC enrichment signatures onto the CD34^+^bulk UMAP. 270, 207 and 184 cells were sorted for the classical, Sumide et al. and EPCR^+^ signature, respectively, of which 15 cells are common to all three signatures. **b** Venn diagrams showing overlap of differentially expressed genes (DEGs) between the different HSC enrichment signatures. For each signature, the number of DEGs between cells corresponding to that signature based on in silico sorting and the background (other) is depicted. **c** Overview of top 25 enriched genes corresponding to the Sumide et al. signature and their rank in the different HSC enrichment signatures.

Inspired by the co-expression pattern of HSC markers evident in our data, we next asked if both levels of information (mRNA and ADT expression) could be used to visualize and compare HSC enrichment strategies via in silico sorting. This bioinformatic approach harnesses ADT expression values to recreate gating strategies used in FACS sorting. Three sorting strategies were compared by this method: (1) The “classical” HSC enrichment scheme (lin^−^CD34^+^CD38^−^CD45RA^−^CD90^+^CD49f^+^)^15^; (2) an HSC enrichment strategy that was recently described to yield a highly purified population of engraftable HSCs from CB (lin^−^CD34^+^CD38^−^CD133^+^GPI-80^+^), which we refer to as the “Sumide et al.” signature^16^ and corresponds well to the GPI-80-based functional enrichment strategy used in this work; and (3) an “EPCR^+^” signature (CD34^+^CD38^−^CD201^+^). An overview of the in silico sorting strategy is presented in Supplementary Fig. 6A, whereby starting from the CD34^+^bulk fraction CD34^+^CD38^−^ cells were gated and then sub-gated via additional positive selection markers of each signature, guided by our flow cytometry experiments (Supplementary Fig. 6a, b) (described in detail in Supplementary Methods). Following in silico sorting, cells corresponding to the three HSC enrichment strategies were projected onto a common UMAP reference frame, revealing considerable similarity between the sort strategies, with all three approaches enriching for cluster 0 cells (Fig. 5a). The majority of DEGs for both the classical (82.94%) and EPCR^+^ (80.12%) signatures were shared with the GPI-80 centered Sumide et al. signature (Fig. 5b), which closely matches our GPI-80-based functional enrichment strategy. Strong correlation between the top enriched genes further corroborated the extensive transcriptomic overlap between cells captured by these different HSC enrichment strategies (Fig. 5c). Altogether, these data suggest that the transcriptomic signature of the engraftable HSC identified in this work is not exclusive to the GPI-80^+^ enrichment strategy, but rather represents a generalizable engraftment signature for FL HSCs irrespective of purification strategy.

## Discussion

Our examination of the molecular programs associated with engraftment capacity serves as a potent resource to empower a broad range of future studies and scientific queries. From the development of strategies that preserve HSC functionality ex vivo, to illuminating the developmental roadmap for the generation of HSCs from PSCs, our data reveal a distinct transcriptional and protein-level program that can be harnessed to fuel potential improvements in these approaches.

To arrive at an in-depth characterization of engraftable FL HSCs, we harnessed the functional HSC enrichment capacity of GPI-80, a marker closely associated with engraftment^16,17^. Despite representing only 2.37% of total CD34^+^ cells, sorting thousands of CD34^+^GPI-80^+^ cells allowed us to perform parallel profiling of this rare population at the functional, transcriptional and surface protein level. After confirming their superior engraftment potential via xeno-transplantation, we mined the transcriptome of these rare cells, resulting in a molecular signature of engraftable FL HSCs at unprecedented resolution.

Given the transcriptionally distinct clusters we discerned within this highly purified population, we sought to understand which of these transcriptomic profiles exhibited the strongest correlation with engraftment potential. By analyzing cluster proportions before and after functional enrichment, we identified several clusters that were significantly enriched, thus corresponding to putative engraftable HSCs.

One such cluster (HSC/MPP cluster 3) was characterized by a prominent unfolded protein response (UPR) signature, represented by an abundance of genes encoding heat shock proteins (*HSPA1A*, *HSPB1B*, *HSPA6*, *HSPB1*). Interestingly, protein quality control has been linked to the ability of HSCs to maintain their undifferentiated status^29–31^. FL HSCs possess the unique capacity to tolerate significant proliferation without impacting multilineage engraftment potential and this has been associated with a heightened DNA damage response in mouse FL versus postnatal HSCs^32^. Similarly, there appears to be a role for the maintenance of proteome integrity in shielding HSC function during expansion in the FL. While bile acids have been described to serve as chaperones alleviating unfolded protein stress in expanding mouse FL HSCs^33^, our data suggest that heat shock proteins may take on this role in the human FL.

The most highly represented cluster upon functional enrichment (HSC/MPP cluster 0) exhibited increased expression of *RGCC, LMNA*, ID genes and members of the AHR pathway, including *TIPARP*, the product of which has been identified as a negative regulator of AHR activity^21,34^. AHR inhibitors such as SR-1 have been described to expand CD34^+^ CB cells^35^ and we and others have linked AHR inhibition to enhanced endothelial-to-hematopoietic transition and HSPC expansion during in vitro differentiation from PSCs^36,37^, highlighting a role for AHR inhibition in HSPC biology. *LMNA* was identified as the second most enriched gene in this cluster and was a part of both KLF10 and KLF13 regulons executing a cluster 0-specific transcriptional program. *LMNA* encodes the nuclear lamina protein Lamin A/C and is expressed in postnatal HSCs where it shows a decline in expression throughout ageing^22,38^. Interestingly, comparing *LMNA* expression between our FL CD34^+^ cells and existing scRNAseq datasets representing postnatal CD34^+^ and HSC-enriched fractions^9,10,39^, revealed more prominent *LMNA* expression in the FL (Supplementary Fig. 2b), suggesting that the decline in *LMNA* expression might begin in utero. Given the superior engraftment potential of FL HSCs compared to postnatal HSCs^6^, this might suggest a role for LMNA in endowing FL HSCs with this remarkable capacity. Consistent with this hypothesis, *LMNA* has been described to be more highly expressed in LT-HSCs than in less potent ST-HSCs in mice^38^, a finding that is reflected in our data by the enrichment of *LMNA* in the GPI-80^+^ compared to the CD34^+^ bulk fraction (Supplementary Table 2). Altogether these findings support a link between *LMNA* expression and HSC functionality.

We also observed enrichment for members of the inhibitor of DNA binding (ID) gene family along the top edge of cluster 0. Expression of *ID1*, *ID2* and *ID3* has been reported higher in HSCs compared to downstream progenitors in CB and reduction in their expression coincides with loss of quiescence and in vivo repopulating capacity^40^. Moreover, ID1 has recently been implicated in controlling the balance between dividing and resting neural stem cells by promoting quiescence^41^. It is tempting to speculate that ID family members could play a similar role in FL HSCs, especially when taking into consideration co-enrichment for genes encoding other factors involved in cell cycle control in this cluster such as *RGCC* or “regulator of cell cycle” and the predominantly quiescent profile of cluster 0 cells. A similar function has been reported for NFE2L2 in mouse HSCs^24^, in addition to its more widely described role as master regulator in the antioxidant response pathway^24,42,43^. The fact that we find this factor regulating expression of a cluster 0-specific transcriptional program in human FL HSCs suggests that NFE2L2 might be involved in balancing quiescence and self-renewal here as well.

Notably, a recent study profiling PSC-derived HSPCs at the single-cell level, identified *ID2* as enriched in what was considered the most naïve, in vitro-derived HSPC fraction, highlighting an encouraging overlap in gene expression with engraftable HSCs in vivo^44^. In this work, the transcriptomic profile of PSC-derived HSPCs was compared to that from FL HSPCs, identifying additional commonalities but also dissimilarities that might suggest how to further improve PSC-based HSC generation. In line with this goal, we believe that our comprehensive characterization of engraftable HSCs and the detailed engraftment signature that cluster 0 represents will be of particular interest to the field to further optimize this process. Moreover, comparison to postnatal HSC-enriched fractions indicated that the expression of several cluster 0 enriched genes was higher in the FL compared to the BM, suggesting that the signature identified in this work specifically represents HSCs at a stage where they display superior engraftment potential. Given that this prenatal stage presents a developmentally more relevant comparator than postnatal HSC sources when it comes to in vitro generation of HSCs starting from PSCs, we consider our dataset especially valuable in pursuit of this goal for regenerative medicine applications.

Beyond in-depth transcriptional characterization, this study also provides linked cell-surface marker expression data of engraftable FL HSCs. Comparing both transcript and protein expression data, we found that ADT read-outs offered complementary insights as they allowed for the identification of marker co-expression that was not readily apparent based on mRNA expression alone. Importantly, ADT but not mRNA expression data suggested that EPCR (CD201) could be used as a cell-surface marker to specifically enrich for cluster 0 cells and thus engraftable FL HSCs, which was confirmed by the superior engraftment potential of CD201^+^ selected cells in transplantation experiments in immunocompromised mice. While this marker has been proposed as a useful addition to existing enrichment strategies to purify FL HSCs^18^, the linked mRNA and ADT data in this work would suggest that EPCR may serve as a viable single enrichment marker for functional FL HSCs.

Using flow cytometry, we further extended our protein-level characterization of FL HSPCs through simultaneous assessment of 21 cell-surface markers and confirmed the expression patterns observed based on ADT expression data as representative across multiple biological replicates of FL. This dataset can be accessed and further interrogated via the following repository (http://flowrepository.org/id/FR-FCM-Z32M). The UMAP representation of the multi-dimensional flow data highlighted a region characterized by co-expression of several prominent HSC enrichment markers, suggesting that it could represent the apex of the hematopoietic hierarchy where engraftable HSCs reside. This interpretation was further supported by the almost exclusive localization of CD201 expression in this region, which we have functionally validated to further enrich for engraftment potential as suggested by its ADT expression profile. These observations are in line with the recent molecular characterization of mouse HSCs where *Procr* (*Epcr*) was shown to be enriched in the most primitive subset of functional long-term repopulating HSCs^7^. While the CITE-seq profiling was performed on a single biological FL sample, which could be seen as a limitation of this study, the confirmation of expression patterns by multiparameter flow cytometry as well as functional validation of the predictions regarding superior engraftment potential of the CD201^+^ fraction resulting from our integrated transcriptomic and cell-surface level expression analysis, highlight the biological accuracy of our dataset and analysis.

Lastly, to illustrate the added value of cell-surface marker expression data linked to transcriptomic data we integrated ADT and mRNA information to compare three well-established HSC enrichment strategies. Through in silico sorting, we gated populations of interest based on ADT expression and compared transcriptomic profiles of the resulting cells. Here, a GPI-80-based enrichment strategy showed very high similarity to enrichment strategies driven by either CD90^+^CD49f^+^ or EPCR^+^ enrichment. This led us to conclude that the engraftment signature that we identify in this work is representative of engraftable FL HSCs irrespective of the markers used during isolation. Using our dataset, the same in silico sorting approach can be harnessed to query other markers without the need to physically sort out cell fractions and subject them to transcriptomic profiling.

The unprecedented resolution at which the engraftable HSC fraction is interrogated in this study, together with the multi-modal profiling of these cells at the functional, transcriptomic and protein-level, makes our in-depth characterization unique among prior studies interrogating HSPCs. We envision that this openly shared resource, which has been made available in an interactive format at https://engraftable-hsc.cells.ucsc.edu, will enable new biological insights into engraftment potential, including how it can be retained during ex vivo culture and how it can be induced to generate functional HSCs from PSCs.

## Methods

### Ethics statement

#### The reported research complies with all relevant ethical regulations

All mouse research complied with the Institutional Animal Care and Use Committee (IACUC) of the Massachusetts General Hospital (MGH) (Protocol #2009N000136). Human fetal liver tissues were obtained from participants who consented in writing to the use of tissues resulting from termination of pregnancy for research. Participants received no compensation.

Human peripheral blood mononuclear cells were obtained from a commercial provider as de-identified discarded human material.

This study was reviewed by the Mass General Brigham Institutional Review Board (IRB Protocol #2016P001106) and was determined to be exempt as it does not constitute human subjects research given its use of de-identified, discarded material.

#### Processing of fetal liver (FL) samples

The stage of the FL samples is indicated as post conception weeks (pcw). The FL sample used for CITE-seq was collected at 21 pcw (sex NA). For the multiparameter flow characterization the following 5 samples were used: FL1 (21 pcw, sex N/A), FL2 (22 pcw, F), FL3 (16 pcw, sex N/A), FL4 (17 pcw, sex N/A), FL5 (17 pcw, F). FL samples were mechanically dissociated into small pieces and incubated in Liver Digest Medium (Fisher Scientific, 17703034) at 37 °C. Mononuclear cells were isolated over a Ficoll gradient (Lymphoprep: Stem Cell Technologies, 7851) prior to separation into CD34^+^ and flowthrough (CD34^−^) cells using magnetic beads (CD34 Microbead Kit: Miltenyi Biotec, 130-046-702).

#### CITE-seq sample preparation

FL cells were thawed and allowed to recover at 37 °C for an hour prior to staining. Cells were blocked with TruStain FcX (BioLegend, 422301) and stained with TotalSeq A antibody mix containing 1ug of each TotalSeq A antibody per condition (BioLegend, see Table S1 for a list of antibodies). Anti-human CD235a-APC antibody (BD Biosciences, 551336) was added to the CD34^−^ fraction. The CD34^+^ fraction was stained with anti-human CD34-APC antibody (BD Biosciences, 555824) and anti-human GPI-80-PE antibody (MBL International, D087-5). All samples were stained with calcein blue (Invitrogen, C34853) for live/dead exclusion and cell populations were sorted (Beckman Coulter MoFlo Astrios) as shown in Fig. S1A prior to loading onto the 10x Genomics platform. Chromium Single-Cell 3**ʹ** Reagent Kit v3 with Feature Barcoding technology for Cell-Surface Protein was used and the recommendations from the manufacturer were followed as specified in 10x Genomics user guide document number CG000185, Rev B.

#### Transplantation experiments

NOD/SCID/*IL2rγ*^*null*^ (NSG) mice (JAX NSG 005557 - NOD.Cg-Prkdc<scid> Il2rg<tm1Wjl>/SzJ) were given sub-lethal irradiation (200 cGy) and cells were injected via the retro-orbital sinus. Cells from the same sorted fractions as used in the CITE-seq experiment were transplanted into NSG mice (14–16 weeks old, F). Five mice were transplanted per condition with 10,000 cells each (4000 cells for the GPI-80+ condition). Engraftment was assessed after 16 weeks by determining the ratio (%) of peripheral blood cells expressing human CD45 versus total CD45 expressing cells. Cells were stained with APC-conjugated anti-human CD45 antibody (BioLegend, 304012) and anti-mouse CD45-PacBlue antibody (BioLegend, 103126). To assess engraftment of the CD34^+^CD201^+^ sorted fraction, six NSG mice (13–15 weeks old, F) were transplanted per condition with 2500 cells each. Engraftment of the different sorted fractions shown in Fig. 3h was checked at weeks 4, 8, 12, 16, and 20 post transplantation and multilineage reconstitution potential was assessed 20 weeks post transplantation by checking for expression of human lineage markers in the human CD45+ fraction of peripheral blood using the following antibodies (1:50 dilution): BV421-conjugated anti-human CD4 antibody (BioLegend, 300532), BV605-conjugated anti-human CD3 antibody (BioLegend, 300460), BV650-conjugated anti-human CD20 antibody (BioLegend, 302336), BV785-conjugated anti-human CD45 antibody (BioLegend, 304048), PE-conjugated anti-human CD33 antibody (BioLegend, 303404), PerCP-Cy5.5-conjugated anti-human CD14 antibody (BioLegend, 325622), APC-conjugated anti-human CD8 antibody (BioLegend, 301049).

#### CITE-seq: transcriptomic analysis

Fastq files were generated and counts extracted from each of the three runs separately for expression libraries and antibody-derived tag (ADT) libraries using bcl2fastq v.2.2 and cellranger v.3.0.2. The expression libraries were mapped to a combination of the human and mouse genome references (GRCh38 and GRCm38), in order to detect both human and mouse cells. The ADT counts were summarized using CITE-Seq-Count v 1.4.2. We used Seurat v.3 to further process the data. The proportion of cells which included both human and mouse genes, was used as a threshold to estimate the empirical doublet rate. As expected based on the 10X Chromium guidelines, the doublet rate was proportional to the density of cell loading, but was found to be half the rate originally calculated. Cells with more than a 25% of reads mapping to mitochondrial genes were filtered out. The transcriptomic and ADT assays were aggregated into an integrated multi-assay analysis. The CD34^−^GYPA^−^ (CD34N-CD235AN) fraction generated 10,253 cells at a depth of 33,334 reads/cell. The CD34^−^GYPA^+^ (CD34N-CD235AP) fraction generated 5,957 cells at a depth of 42,728 reads/cell. The CD34^+^ bulk (CD34P-Bulk) fraction generated 10,082 cells at a depth of 29,308 reads/cell. The GPI-80^+^ (CD34N-GPI80P) fraction generated 8,529 cells at a depth of 33,017 reads/cell. Quality control was performed with the singleCellTK package^45^. We observed 9904, 4901, 9870.5, 10187 median UMIs, and 2681, 1401, 2808, 2923 median genes detected for fractions CD34^−^GYPA^−^, CD34^−^GYPA^+^, CD34^+^ bulk, and GPI-80^+^, respectively (Supplementary Fig. 7). The median levels of contamination estimated by DecontX was <1% for all fractions^46^. For the combined analysis, the four samples were merged and then normalized using SCTransform. Cell cycle scores were generated using the default S and G2M gene lists in Seurat derived from Tirosh et al.^47^. Scores for S and G2M were assigned using the CellCycleScoring function in Seurat and each cell was assigned a cell cycle Phase: either G1(G0), G2/M or S using the same function. These scores were then regressed out along with percent mitochondrial content while normalizing the data. To cluster cells principal component analysis (PCA) was performed and the top 30 principal components (PCs) were used as input for the Louvain clustering algorithm (resolution 0.75). For 2D visualization purposes the dimension reduction algorithm UMAP was ran on the top 30 PCs using default settings. Differential expression tests were done with MAST^48^. The same analysis pipeline was used to analyze the recently published fetal liver cell atlas^14^. In addition, the top 20 markers for each of the cell types described in Popescu et al. were computed using a Wilcoxon test and ranked by Log-fold-change. The corresponding gene signatures for each cell type were used to score their enrichment in our four fractions, enabling the annotation of our dataset based on the modules extracted from the Fetal Liver Atlas. The correspondence between cell types present in both datasets was also validated by integrating both datasets using the pipeline previously mentioned and a harmonization step to correct batch effects. A cluster representing cells with high mitochondrial content and mixed lineage identities was excluded from downstream analysis. Six clusters identified in the progenitor compartment were grouped into one HSC/MPP cluster for the representation in Fig. 1a. For the separate analysis of the CD34 + HSC/MPP compartment, clusters with a majority of CD34^+^ cells were extracted from the merged analysis of all four fractions and re-clustered in an analogous manner as described above with resolution = 0.5. From this subset, cells were separated by original identity into a GPI80^+^ fraction and a CD34^+^ bulk fraction for downstream analyses, maintaining the cluster identities established upon re-clustering of the CD34 + HSC/MPP compartment.

#### Trajectory analysis

Following the transcriptomic analysis described above of the four combined fractions, we performed trajectory analysis in R version 3.6^49^ using a trajectory analysis package: Monocle version 3.2^27,50–54^. We used the UMAP embeddings/coordinates from the transcriptomic analysis to perform trajectory analysis. Branches of the trajectory were created using clustering information and cell lineage determinations from the transcriptomic analysis. Each branch consists of the HSC/MPP populations and the clusters from one of the four lineages (erythroid cells, lymphoid B cells, T and NK cells, and myeloid cells), which were analyzed separately as four separate trajectories (and therefore do not have the exact same root node). A root node was selected for each branch from within the HSC/hematopoietic progenitor cell populations using the unbiased method provided in the Monocle 3 documentation (https://cole-trapnell-lab.github.io/monocle3/). The cells in each branch were assigned a pseudotime value with the Monocle 3 pseudotime ordering algorithms and the root node set as time zero. Gene expression changes over pseudotime were investigated in each branch and selected lineage markers were highlighted using heatmaps.

#### Comparison to postnatal datasets

In order to investigate *LMNA* expression in prenatal HSPCs versus postnatal HSPCs we obtained raw count data from publicly available datasets^7,8,31^. These three datasets were merged with the analysis of the CD34^+^ bulk fraction and then processed using the same steps described for the transcriptomic analysis of our four fractions combined in order to compare expression of *LMNA*. To specifically compare expression of cluster 0 DEGs between HSC-enriched fractions, we compared gene expression in our GPI-80+ fraction with that of postnatal CD49f + gated index-sorted cells that underwent single-cell transcriptomic profiling^9^.

#### Gene regulatory network (GRN) analysis

To further dissect the transcriptional signature of the GPI-80^+^ HSC/MPP compartment, the Single-cell Regulatory Network Inference and Clustering (SCENIC)^23^ pipeline was applied to our SCTransform normalized data. This pipeline first identifies regulons (sets of genes regulated as a unit) that are co-expressed with transcription factors in our dataset, then selects only the regulons with significant motif enrichment, and finally uses their AUCell algorithm to assign a score to each regulon for each cell. UMAP embeddings/coordinates from our transcriptomic analysis were used to display the AUC regulon scores and a Regulon Specificity Score (RSS)^55^ was used to identify regulons important in each of the GPI-80^+^ clusters.

#### Mouse embryonic stem cells (mESCs)

mESCs from Nkx2-1^mCherry^ mice^56^ were spiked into fractions prepped for CITE-Seq to control for background staining from the TotalSeq A antibodies. Undifferentiated mESCs were maintained on a feeder layer of mitotically inactivated mouse embryonic fibroblasts (MEFs) in serum-containing media consisting of Dulbecco’s modified Eagle medium (Life Technologies, 11995-073) with 15% fetal bovine serum (Thermo Fisher Scientific, NC0712155), 200 mM l-glutamine (Invitrogen, 25030-164) and 100 μg/ml Primocin (Thermo Fisher Scientific, NC9392943), supplemented with LIF-containing conditioned media (Millipore, ESG1106) at 1 U/ml. mESCs were passaged as needed when cultures reached appropriate confluency using 0.05% trypsin (Invitrogen, 25300-120).

### CITE-seq: ADT processing and analysis

#### ADT data transformation and background removal

Prior to sorting, cells were stained with a panel of oligo-tagged antibodies (Table S1) so that the antibody-derived tags (ADTs) corresponding to the cell-surface markers present on each cell would also be captured in the sequencing data. Non-specific staining using oligo-tagged antibodies was minimal and was corrected based on background staining levels detected in mouse cells (mESCs) that were spiked into the profiled fractions to account for noise. ADT centered-log-ratio (CLR) transformation and background removal was performed as reported with adaptations^19^. Specifically, 8735 CD34^+^bulk cells and 207 mouse cell spike-ins, raw ADT count data were CLR-transformed for each of the ADTs, using the function NormalizeData with normalization.method = “CLR” and margin = 1 in Seurat V3.2.1. The value at one standard deviation greater than the average CLR-transformed ADT counts from mouse cells were defined as the background cutoff and were subtracted from the 8735 human CD34^+^bulk cells. The same procedure was applied to 7235 GPI-80^+^ cells and 188 mouse cell spike-ins.

#### Dimension reduction using UMAP

Log-normalized mRNA data and CLR- transformed ADT data were grouped into 10 bins and colored according to their feature expression levels, respectively. For CLR-transformed ADT data, the top 1 percent feature values were labeled outlier and removed by specifying max.cutoff = “q99” in FeaturePlot function in Seurat V3.

#### In silico gating strategy

For in silico sorting purposes, cells were first gated based on CD34^+^CD38^−^ criteria and subsequently underwent signature-specific gating (schematic overview presented in Supplementary Fig. 6a). This process was guided by the average percentages obtained for the different marker combinations in flow cytometry experiments on 5 individual FL samples to ensure that the in silico gating reflected populations that would be obtained via analogous FACS sorting (Supplementary Fig. 6a, b). As the CD38 oligo-tagged antibody in our CITE-seq panel did not result in ADT signal due to technical issues specific to this antibody, we based CD38^−^ selection in the first gating on mRNA values for CD38 (See Supplementary Fig. 6c, d for surface level CD38 expression and its correlation with CD34 and GPI-80 surface level expression). While in this study we used a GPI-80 selection step to enrich for engraftment potential resulting in the profiling of 7235 GPI-80^+^ cells and establishment of a detailed engraftable FL HSC signature, an oligo-tagged GPI-80 antibody was not included in our CITE-seq panel. Given the poor correlation between GPI-80 protein expression and the corresponding mRNA expression of its encoding gene *VNN2* (Supplementary Fig. 6e), we consulted the extended transcriptomic signature of the GPI-80^+^ cell population to derive a GPI-80 module score and approximate GPI-80 protein expression in the CD34^+^CD38^−^ gated population. This module score was obtained by generating an AddModuleScore based on the top 30 enriched genes within the GPI-80^+^ HSC/MPP fraction and used to retro actively label putative GPI-80 expressing cells within the CD34^+^CD38^−^ population based on how strongly their expression profile resembled GPI-80^+^ sorted cells^47^. The module scores were calculated using the AddModuleScore function in Seurat V3.2.1 with features being the top 30 positively enriched genes within the GPI-80^+^ HSC/hematopoietic progenitor cell fraction, assay = “SCT”, and default parameters. When displaying the resulting continuous “GPI-80 module score” values versus CD34 + ADT values, we found a distribution that strongly resembled FACS data (Supplementary Fig. 6f). To approximate what was sorted for the CITE-seq analysis based on GPI-80 cell-surface marker expression, the top 2.37% of CD34^+^ cells positive for GPI-80 identity based on this GPI-80 score were considered (corresponding to 3% of CD34^+^CD38^−^ gated cells, Supplementary Fig. 6a). Those GPI-80 score^+^ cells that were also CD133^+^, were gated to reflect the Sumide et al. signature for comparison to the “classical” (CD90^+^CD49f^+^) and “EPCR^+^” (CD201^+^) signature, which were both gated based on ADT expression values.

For two-dimensional density plots showing in silico gating thresholds. Density was estimated using two-dimensional kernel density estimation with an axis-aligned bivariate normal kernel, evaluated on a square grid. Function kde2d in R package MASS V7.3-51.6 was used with number of grid points in each direction equals 500 (*n* = 500).

### Processing of peripheral blood mononuclear cells (PBMCs)

PBMCs from a 65-year-old individual (M) were used as a control for the flow cytometry experiments. Mononuclear cells were isolated from a commercially available healthy blood leukapheresis pack (New York Biologics Inc) over a Ficoll gradient (Cytiva, 17144003).

### Multi-dimensional flow cytometry

All antibodies were purchased from commercial vendors (Table S5) and were pre-titrated using PBMC and FL cells. For the 22-color analysis, PBMC and FL cells were thawed, washed, blocked with Human FcBlock (BioLegend, 422301) and stained with Live-Dead Blue amine dye (Thermo Fisher, L34961). A cocktail of antibodies was prepared fresh and supplemented with Monocyte Blocker (BioLegend, 426102) and Brilliant Buffer Plus (BD Biosciences, 566385). Cells were stained in the dark at 4 °C and washed twice. A sub-panel of brightly expressed markers was assessed as a separate stain to serve as a fluorescence-minus-multiple control for further data interpretation. PBMC cells and single-stain Ultracomp beads (Thermo Fisher, 01-2222-42) were stained to provide single-stain spectral controls. Cells and single-stain controls were analyzed on a 5-laser Aurora spectral flow cytometry (Cytek Biosciences) and raw fluorescence data from 64 channels were unmixed using ordinary least square algorithm in Spectroflo v2 (Cytek Biosciences).

### Fluorescence cytometry data analysis

Data analysis pipelines were built using cloud-based OMIQ analysis platform (Omiq). Briefly, single-cell data were asinh transformed (cofactor 6000) and 100,000 (as defined by least size FL sample) live single-cell events from each sample were selected for analysis. All fluorescence data (excluding live-dead dye staining intensity) from 5 FL samples and 1 PBMC sample were projected into two-dimensional space with UMAP algorithm^54^ (neighbors = 15; minimum distance = 0.4; learning rate = 1; epochs = 200). For analysis, intensities of each fluorescence parameter were overlaid on UMAP maps to represent expression levels. Same dataset was clustered with Phenograph^27^ (*k* = 20, distance metric = euclidean) and clusters were color-coded and overlaid over UMAP maps. Phenograph clusters were also plotted as heatmaps to represent cell abundance over multiple clusters and median fluorescence intensity across multiple surface protein markers.

### Quantification and statistical analysis

Specifics about the statistical tests and replicates used in each experiment are available in the figure legends or specified in the text. The *p*-value threshold to determine significance was set at *p* = 0.05. *p*-value annotations on graphs are as follows: *p < 0.05, **p < 0.01, ****p < 0.0001 and are based on a one- or two-sided analysis of variance (ANOVA) test with Tukey’s multiple comparisons adjustment. Data for quantitative experiments is typically represented as the mean with error bars representing the standard error of the mean, as specified in the figure legends.

### Reporting summary

Further information on research design is available in the Nature Research Reporting Summary linked to this article.

## Supplementary information

Reporting Summary (updated version 20220119)

Supplementary Information

Supplementary Table 2

Supplementary Table 3

## Data Availability

The raw CITE-seq data generated in this study has been deposited in the GEO database under accession number GSE160251. The CITE-seq data generated in this study is also available through the following interactive browser [https://engraftable-hsc.cells.ucsc.edu]. The multiparameter flow cytometry data generated in this study (unmixed fluorescence cytometry dataset) has been deposited in the FlowRepository database [http://flowrepository.org/id/FR-FCM-Z32M]. The human genome (GRCh38) sequence data used in this study is available through the Ensembl genome browser [http://ftp.ensembl.org/pub/release-104/fasta/homo_sapiens/dna/]. The mouse genome (GRCm38) sequence data used in this study is available through the Ensembl genome browser [http://ftp.ensembl.org/pub/release-104/fasta/mus_musculus/dna/]. Source data are provided with this paper.

## Source data

Source Data File
Reporting Summary (previous version)

## Code availability

The code used in this work is available at [https://github.com/CReM-BU/Vanuytsel_hPSC^57^]. Multiparameter flow cytometry code (unmixed fluorescence cytometry dataset with integrated UMAP and Phenograph parameters) is available at [http://flowrepository.org/id/FR-FCM-Z32M].
