# Supplementary material for: Multi-modal profiling of human fetal liver hematopoietic stem cells reveals the molecular signature of engraftment
Source: Nat Commun. 2022 Mar 1;13:1103. doi: 10.1038/s41467-022-28616-x (PMC8888592; doi:10.1038/s41467-022-28616-x)
Supplement: Supplementary file 2 — Supplementary Information [file 41467_2022_28616_MOESM2_ESM.pdf]

INVENTORY OF SUPPORTING INFORMATION

Supplementary Figure Legends
Supplementary Figures
Supplementary Table Legends
Supplementary Tables
Supplementary References

SUPPLEMENTARY FIGURE LEGENDS

Supplementary Fig. 1. Experimental outline and gene expression changes over pseudotime

a Overview of fractions isolated from a FL sample and how they were subdivided in individual fractions to be run on the 10X Genomics platform for CITE-seq analysis and simultaneous transplantation experiments. Like the $CD34^{+}$ fraction, the $CD34^{-}$ flowthrough was first gated to select live cells prior to GYPA gating. At the time of sorting this fraction contained 87% $CD34^{-}$ cells and 13% $CD34^{+}$ cells. The $CD34^{+}$bulk cells that were live gated after $CD34^{+}$ magnetic bead selection contained 97.4% $CD34^{+}$ cells and 2.64% $CD34^{-}$ cells. The GPI-$80^{+}$$CD34^{-}$ cells captured in the GPI-80 sort gate represent macrophages. This population (349 cells) was not considered for the in-depth profiling of the GPI-80^{+} fraction which starts from cells captured within the $CD34^{+}$ HSC/MPP fraction in **Figure 1a**. **b-e** Pseudotime trajectories per hematopoietic lineage (left) and heatmaps illustrating gene expression changes over pseudotime for HSC/MPP and key lineage markers (right) for the erythroid (**b**), lymphoid (B cell) (**c**), lymphoid (T/NK cell) (**d**) and myeloid (**e**) lineage. Interestingly, $GYPA^{+}$ (CD235a) sorting also appeared to enrich for cells with a signature of B cells (**a,c**). While reports of *GYPA* expression in B cells exist[1], we did not observe CD235a staining on B cells in subsequent validation experiments on 5 additional FL samples using our multi-parameter flow panel (**Figure 4c**). A difference in concentration of the CD235a antibody between this sort and the multi-parameter flow cytometry experiment might explain the increased likelihood of picking up non-specific or low levels of CD235a signal during the initial sort, resulting in co-purification of B cells in the $GYPA^{+}$ gate.

Supplementary Fig. 2. Dissecting the molecular signature of HSC/MPP cluster 0 cells.

a Dot plot illustrating the distribution of a selection of genes enriched in the GPI-80^{+} compared to $CD34^{+}$ bulk HSCs/MPPs. Scaled average expression is shown. 'Percent expressed' represents the fraction of cells having non-zero expression values. **b** Comparison of *LMNA* expression in prenatal HSPCs versus postnatal HSPCs. We

compared CD34+ bulk FL HSC/MPPs from this study with existing scRNAseq data sets representing postnatal CD34+ fractions[2-4] in terms of *LMNA* expression. Scaled average expression is shown in the dot plot. ‘Percent expressed’ represents the fraction of cells having non-zero expression values. **c** Comparison of expression between HSC-enriched fractions in the FL (this study, GPI-80+ fraction) and BM (Velten et al., 2017[4], CD49f+ gated) for a selection of genes enriched in HSC/MPP cluster 0. Scaled average expression is shown. ‘Percent expressed’ represents the fraction of cells having non-zero expression values. **d** Regulon Specificity Score (RSS) plot highlighting the top 5 regulons with the highest specificity for HSC/MPP cluster 0 within the GPI-80+ fraction. **e** Heatmaps illustrating row-scaled average expression of the genes within a regulon for NFE2L2, KLF13 and KLF10 regulons. The scale bar shows row-scaled average expression from 0-Max.

Supplementary Fig. 3. Dissecting the molecular signature of cells belonging to HSC/MPP cluster 2 and 3.

a Overview of cell cycle distribution for the different HSC/MPP clusters within the GPI-80+ fraction (left) and the CD34+bulk versus GPI-80+ fraction (right). UMAP representations of cell cycle phase distribution and S and G2/M scores are illustrated below. The scale bar shows scaled expression from 0-Max. **b** EnrichR (https://maayanlab.cloud/Enrichr) read-out (-log(p-value)) for HSC/MPP cluster 2 in the GPI-80+ fraction. Black bars indicate when the FDR is significant with a score of <0.05.

c Regulon Specificity Score (RSS) plot highlighting the top 5 regulons with the highest specificity for HSC/MPP cluster 2 within the GPI-80+ fraction. **d** EnrichR (https://maayanlab.cloud/Enrichr) read-out (-log(p-value)) for HSC/MPP cluster 3 in the GPI-80+ fraction. Black bars indicate when the FDR is significant with a score of <0.05.

e Regulon Specificity Score (RSS) plot highlighting the top 5 regulons with the highest specificity for HSC/MPP cluster 3 within the GPI-80+ fraction.

Supplementary Fig. 4. Integration of mRNA and (antibody-derived tag) ADT data identifies CD201 as a marker enriching for highly functional HSCs.

a Comparison of mRNA and ADT expression for a selection of HSC markers in the $CD34^{+}$bulk fraction. The scale bar shows scaled expression from 0-Max. **b** Comparison of mRNA and ADT expression for a selection of HSC markers in the GPI-80^{+} fraction. The scale bar shows scaled expression from 0-Max. **c-e** Transcriptomic cluster composition (%) within the $CD34^{+}$ bulk fraction upon stepwise enrichment for cell surface marker expression of CD90 (**c**), CD49f (**d**) and ENG (**e**). The x-axis is divided in segments showing the indicated top x% of expression for each marker. **f** Longitudinal engraftment analysis illustrating superior engraftment of $CD34^{+}CD201^{+}$ compared to all other fractions, including $CD34^{+}$GPI-80^{+} cells. Six NSG mice were transplanted per condition with 2500 cells each and engraftment was assessed by determining the percentage (%) of peripheral blood cells expressing human CD45 at weeks 4, 8, 12, 16 and 20. The mean is shown with error bars representing the standard error of the mean. Two-way analysis of variance (ANOVA) was performed with Tukey test to account for multiple comparisons and obtain adjusted p-values: For the 16-week time point, p=0.0014 (**) for $CD34^{+}CD201^{+}$ vs. $CD34^{+}$GPI-80^{+} and p<0.0001 for $CD34^{+}CD201^{+}$ compared to all other conditions. For the 20-week time point, p<0.0001 (****) for $CD34^{+}CD201^{+}$ compared to all other conditions. Source data are provided as a Source Data file. **g** Demonstration of multi-lineage repopulation in mice engrafted with human $CD34^{+}CD201^{+}$ and $CD34^{+}$GPI-80^{+} cells. The average percentages (n=6) of human $CD45^{+}$ peripheral blood cells that stain positive for a given lineage marker are shown. Source data are provided as a Source Data file.

Supplementary Fig. 5. Multi-dimensional flow cytometric characterization across multiple FL samples validates (antibody-derived tag) ADT expression patterns.

a Phenograph analysis showing the distribution of clusters identified in **Figure 4d** over the different FL samples as well as PBMCs. As expected, certain Phenograph clusters were only represented in the PBMC sample as they contained the more mature cell populations. Heat map color indicates percentage of cells per sample allocated to each cluster (the darker the green, the higher the percentage). **b** Phenograph analysis

representing clusters specific to the $CD34^+$ FL cells. The different cell surface markers assessed in this panel are shown on top. Heat map color indicates median fluorescence intensity of each marker expression per cluster and is normalized per column/marker (the darker the green, the higher the median fluorescence intensity). **c** Overview of the contribution of each FL sample to the combined data set.

Supplementary Fig. 6. In silico sorting strategy.

a Outline of *in silico* gating steps as guided by flow cytometry results. The left column shows the initial $CD34^+CD38^-$ gating step. The middle column shows the signature-specific gating including the percentages of cells that were gated based on ADT expression data (and GPI-80 score, see Supplementary Methods section). The right column shows representative flow plots for a FL sample as well as the average percentage of cells in each of the gates based on flow cytometric analysis of 5 individual FL samples (<x%>, n=5). **b** Percentage of cells in each gate based on flow cytometric analysis of 5 individual FL samples. The mean is shown with error bars representing the standard error of the mean (n=5). Source data are provided as a Source Data file. **c** Flow plot illustrating the distribution of CD38 surface expression within the $CD34^+$ fraction of the FL (left) and the GPI-80^+ events in either the $CD34^+CD38^-$ gated fraction (middle) or the $CD34^+CD38^+$ gated fraction (right) for a representative FL sample. **d** Quantification of percentage $CD38^+$ cells in the $CD34^+$ fraction by flow cytometry. Source data are provided as a Source Data file. The mean is shown with error bars representing the standard error of the mean (n=5). **e** VNN2 mRNA expression in the GPI-80^+ fraction. **f** Flow cytometry data (left) compared to a similar representation of the CITE-seq data (right) based on ADT expression for CD34 (y-axis) and the GPI-80^+ score (x-axis) (see Methods). One outlier cell with very high CD34 ADT expression (> 6) was excluded from the CITE-seq data (right).

Supplementary Fig. 7. Data quality assessment of CITE-seq experiment.

a Violin plots showing the total number of gene counts per cell for the fractions $CD34^-$ $GYPA^-$, $CD34^-GYPA^+$, $CD34^+$ bulk, and GPI-80^+. Red dotted lines indicate medians. **b** Violin plots showing the total number of detected genes per cell for the four profiled FL

fractions. **c** Violin plots showing the levels of contamination estimated by DecontX for the four profiled FL fractions.

Figure S1

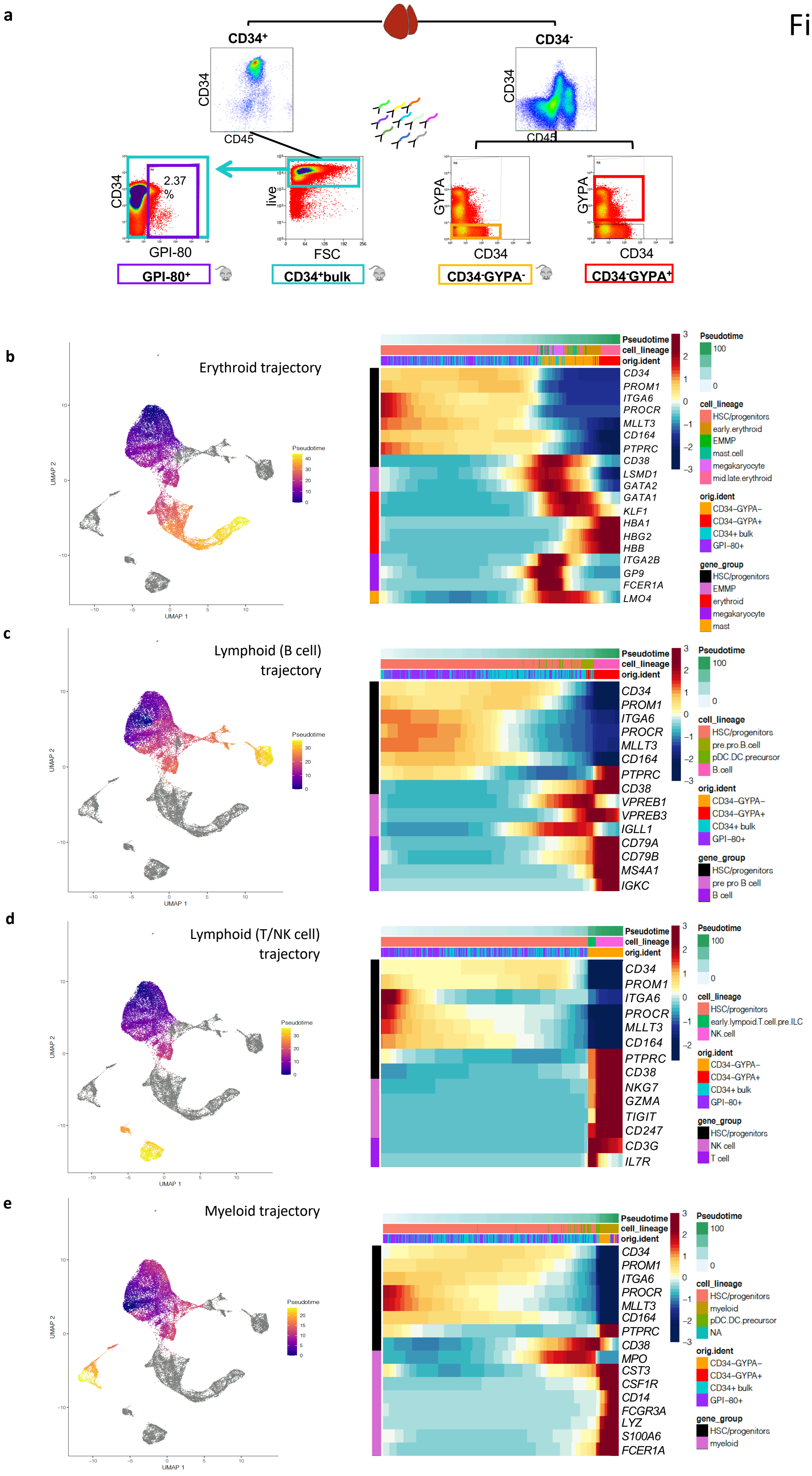
a
CD34+
CD34-
CD34
CD45
2.37 %
GPI-80
live
FSC
GYPA
CD34
GPI-80+
CD34+bulk
CD34-GYPA-
CD34-GYPA+
b
Erythroid trajectory
Pseudotime
cell_lineage
orig.ident
CD34
PROM1
ITGA6
PROCR
MLLT3
CD164
PTPRC
CD38
LSMD1
GATA2
GATA1
KLF1
HBA1
HBG2
HBB
ITGA2B
GP9
FCER1A
LMO4
HSC/progenitors
early.erythroid
EMMP
mast.cell
megakaryocyte
mid.late.erythroid
CD34−GYPA−
CD34−GYPA+
CD34+ bulk
GPI−80+
gene_group
erythroid
mast
c
Lymphoid (B cell) trajectory
VPREB1
VPREB3
IGLL1
CD79A
CD79B
MS4A1
IGKC
pre.pro.B.cell
pDC.DC.precursor
B.cell
pre pro B cell
B cell
d
Lymphoid (T/NK cell) trajectory
NKG7
GZMA
TIGIT
CD247
CD3G
IL7R
early.lympoid.T.cell.pre.ILC
NK.cell
NK cell
T cell
e
Myeloid trajectory
MPO
CST3
CSF1R
CD14
FCGR3A
LYZ
S100A6
FCER1A
myeloid
NA
UMAP 1
UMAP 2

Figure S2

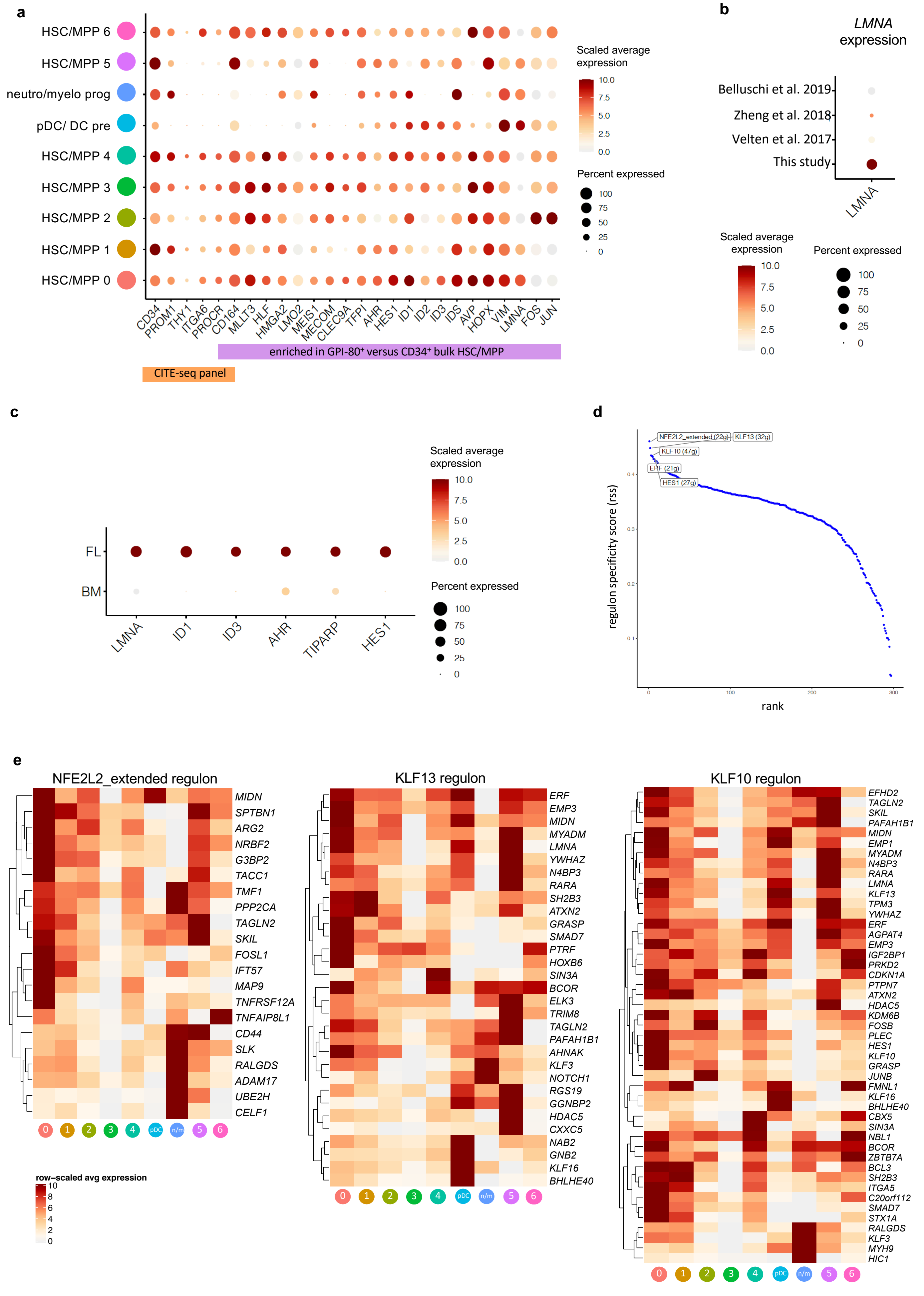
a
HSC/MPP 6
HSC/MPP 5
neutro/myelo prog
pDC/ DC pre
HSC/MPP 4
HSC/MPP 3
HSC/MPP 2
HSC/MPP 1
HSC/MPP 0
CD34 PROM1 THY1 ITGA6 PROCR CD164 MLLT3 HLF HMGA2 LMO2 MEIS1 MECOM CLEC9A TFPI AHR HES1 ID1 ID2 ID3 IDS AVP HOPX VIM LMNA FOS JUN
enriched in GPI-80⁺ versus CD34⁺ bulk HSC/MPP
CITE-seq panel
Scaled average expression
10.0
7.5
5.0
2.5
0.0
Percent expressed
100
75
50
25
0
b
LMNA expression
Belluschi et al. 2019
Zheng et al. 2018
Velten et al. 2017
This study
LMNA
Scaled average expression
10.0
7.5
5.0
2.5
0.0
Percent expressed
100
75
50
25
0
c
FL
BM
LMNA ID1 ID3 AHR TIPARP HES1
Scaled average expression
10.0
7.5
5.0
2.5
0.0
Percent expressed
100
75
50
25
0
d
NFE2L2_extended (22g)
KLF13 (32g)
KLF10 (47g)
ERF (21g)
HES1 (27g)
regulon specificity score (rss)
0.4
0.3
0.2
0.1
0
100
200
300
rank
e
NFE2L2_extended regulon
MIDN SPTBN1 ARG2 NRBF2 G3BP2 TACC1 TMF1 PPP2CA TAGLN2 SKIL FOSL1 IFT57 MAP9 TNFRSF12A TNFAIP8L1 CD44 SLK RALGDS ADAM17 UBE2H CELF1
KLF13 regulon
ERF EMP3 MIDN MYADM LMNA YWHAZ N4BP3 RARA SH2B3 ATXN2 GRASP SMAD7 PTRF HOXB6 SIN3A BCOR ELK3 TRIM8 TAGLN2 PAFAH1B1 AHNAK KLF3 NOTCH1 RGS19 GGNBP2 HDAC5 CXXC5 NAB2 GNB2 KLF16 BHLHE40
KLF10 regulon
EFHD2 TAGLN2 SKIL PAFAH1B1 MIDN EMP1 MYADM N4BP3 RARA LMNA KLF13 TPM3 YWHAZ ERF AGPAT4 EMP3 IGF2BP1 PRKD2 CDKN1A PTPN7 ATXN2 HDAC5 KDM6B FOSB PLEC HES1 KLF10 GRASP JUNB FMNL1 KLF16 BHLHE40 CBX5 SIN3A NBL1 BCOR ZBTB7A BCL3 SH2B3 ITGA5 C20orf112 SMAD7 STX1A RALGDS KLF3 MYH9 HIC1
0 1 2 3 4 pDC n/m 5 6
row-scaled avg expression
10
8
6
4
2
0

Figure S3

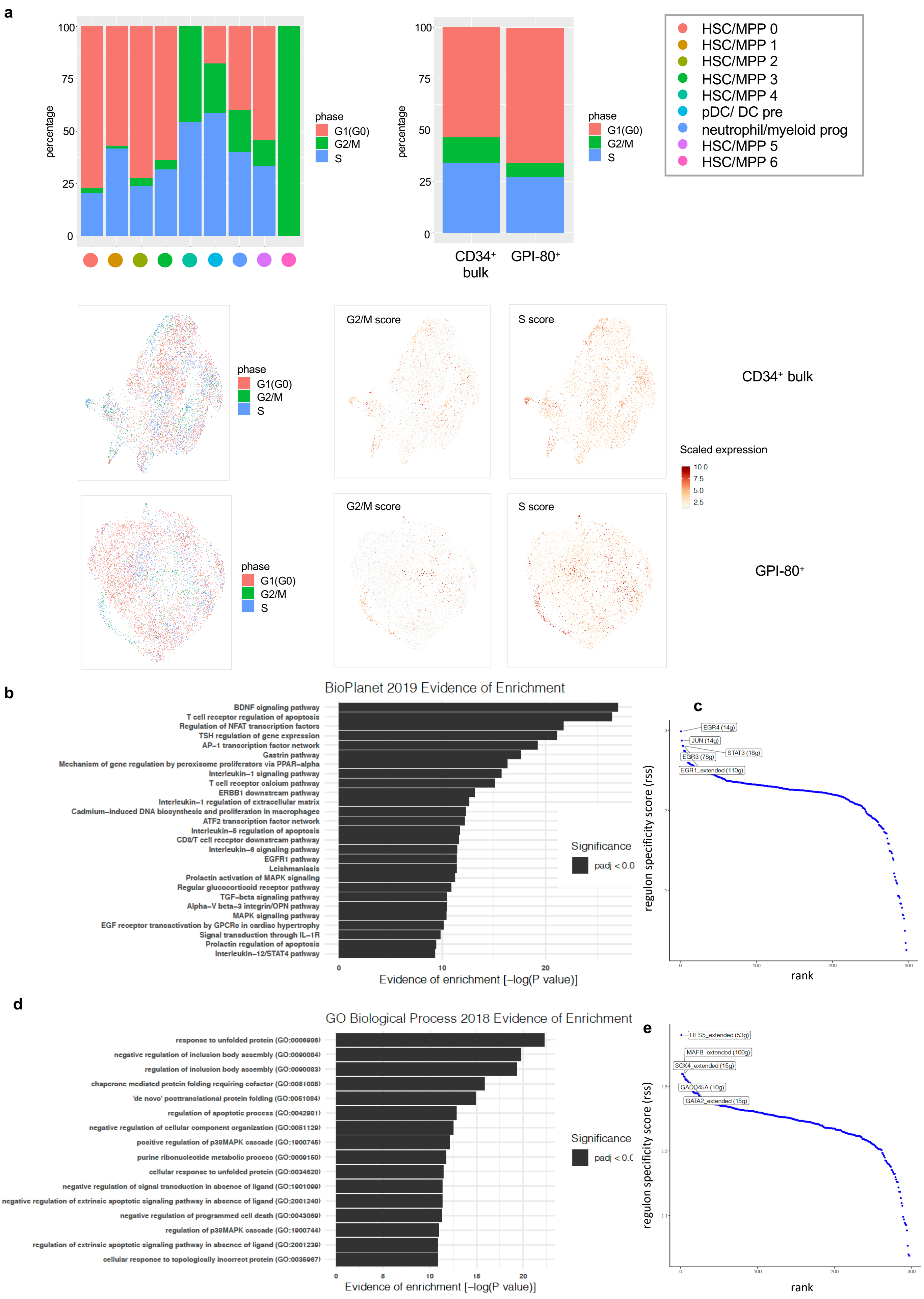
a
100
75
50
25
0
percentage
phase
G1(G0)
G2/M
S
CD34⁺ bulk
GPI-80⁺
HSC/MPP 0
HSC/MPP 1
HSC/MPP 2
HSC/MPP 3
HSC/MPP 4
pDC/ DC pre
neutrophil/myeloid prog
HSC/MPP 5
HSC/MPP 6
G2/M score
S score
CD34⁺ bulk
Scaled expression
10.0
7.5
5.0
2.5
GPI-80⁺
b
BioPlanet 2019 Evidence of Enrichment
BDNF signaling pathway
T cell receptor regulation of apoptosis
Regulation of NFAT transcription factors
TSH regulation of gene expression
AP−1 transcription factor network
Gastrin pathway
Mechanism of gene regulation by peroxisome proliferators via PPAR−alpha
Interleukin−1 signaling pathway
T cell receptor calcium pathway
ERBB1 downstream pathway
Interleukin−1 regulation of extracellular matrix
Cadmium−induced DNA biosynthesis and proliferation in macrophages
ATF2 transcription factor network
Interleukin−5 regulation of apoptosis
CD8/T cell receptor downstream pathway
Interleukin−6 signaling pathway
EGFR1 pathway
Leishmaniasis
Prolactin activation of MAPK signaling
Regular glucocorticoid receptor pathway
TGF−beta signaling pathway
Alpha−V beta−3 integrin/OPN pathway
MAPK signaling pathway
EGF receptor transactivation by GPCRs in cardiac hypertrophy
Signal transduction through IL−1R
Prolactin regulation of apoptosis
Interleukin−12/STAT4 pathway
Significance
padj < 0.0
0
10
20
Evidence of enrichment [−log(P value)]
c
EGR4 (14g)
JUN (14g)
STAT3 (18g)
EGR3 (78g)
EGR1_extended (110g)
regulon specificity score (rss)
rank
d
GO Biological Process 2018 Evidence of Enrichment
response to unfolded protein (GO:0006986)
negative regulation of inclusion body assembly (GO:0090084)
regulation of inclusion body assembly (GO:0090083)
chaperone mediated protein folding requiring cofactor (GO:0051085)
'de novo' posttranslational protein folding (GO:0051084)
regulation of apoptotic process (GO:0042981)
negative regulation of cellular component organization (GO:0051129)
positive regulation of p38MAPK cascade (GO:1900745)
purine ribonucleotide metabolic process (GO:0009150)
cellular response to unfolded protein (GO:0034620)
negative regulation of signal transduction in absence of ligand (GO:1901099)
negative regulation of extrinsic apoptotic signaling pathway in absence of ligand (GO:2001240)
negative regulation of programmed cell death (GO:0043069)
regulation of p38MAPK cascade (GO:1900744)
regulation of extrinsic apoptotic signaling pathway in absence of ligand (GO:2001239)
cellular response to topologically incorrect protein (GO:0035967)
Significance
padj < 0.0
0
5
10
15
20
Evidence of enrichment [−log(P value)]
e
HES5_extended (53g)
MAFB_extended (100g)
SOX4_extended (15g)
GADD45A (10g)
GATA2_extended (15g)
regulon specificity score (rss)
rank

Figure S4

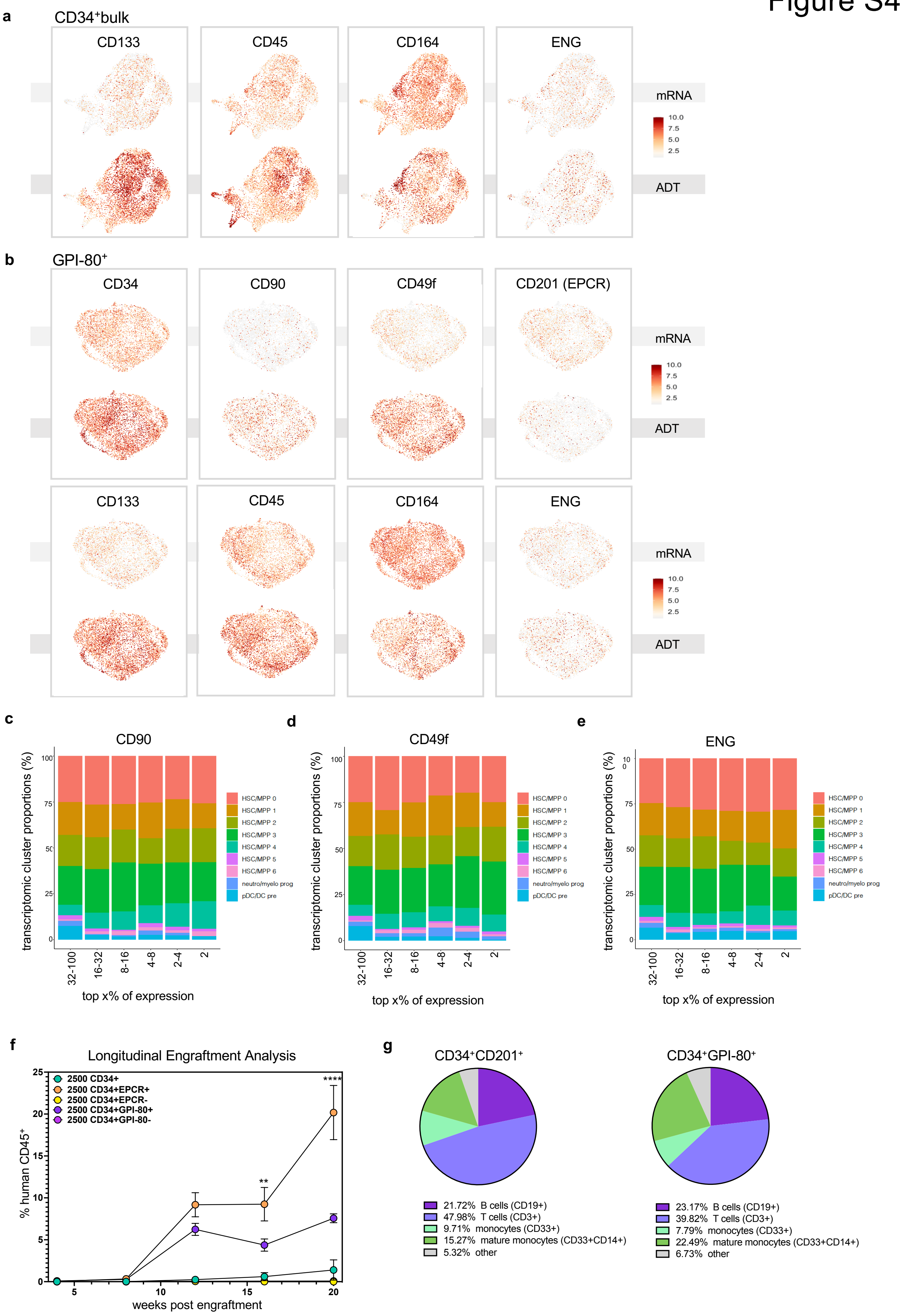

a
CD34+bulk
CD133
CD45
CD164
ENG
mRNA
ADT
10.0
7.5
5.0
2.5
b
GPI-80+
CD34
CD90
CD49f
CD201 (EPCR)
CD133
CD45
CD164
ENG
mRNA
ADT
c
CD90
d
CD49f
e
ENG
transcriptomic cluster proportions (%)
100
75
50
25
0
HSC/MPP 0
HSC/MPP 1
HSC/MPP 2
HSC/MPP 3
HSC/MPP 4
HSC/MPP 5
HSC/MPP 6
neutro/myelo prog
pDC/DC pre
32-100
16-32
8-16
4-8
2-4
2
top x% of expression
f
Longitudinal Engraftment Analysis
2500 CD34+
2500 CD34+EPCR+
2500 CD34+EPCR-
2500 CD34+GPI-80+
2500 CD34+GPI-80-
% human CD45+
weeks post engraftment
**
****
g
CD34+CD201+
21.72% B cells (CD19+)
47.98% T cells (CD3+)
9.71% monocytes (CD33+)
15.27% mature monocytes (CD33+CD14+)
5.32% other
CD34+GPI-80+
23.17% B cells (CD19+)
39.82% T cells (CD3+)
7.79% monocytes (CD33+)
22.49% mature monocytes (CD33+CD14+)
6.73% other

Figure S5

a

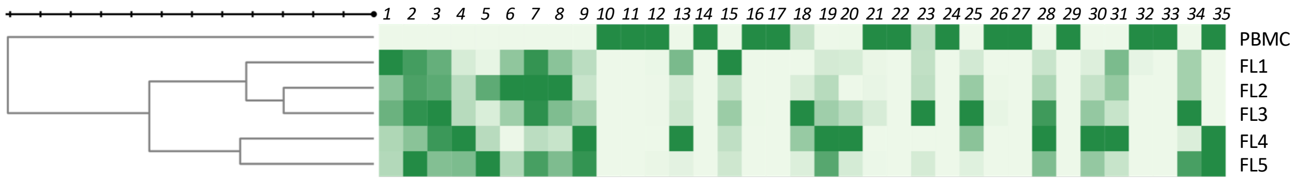

b

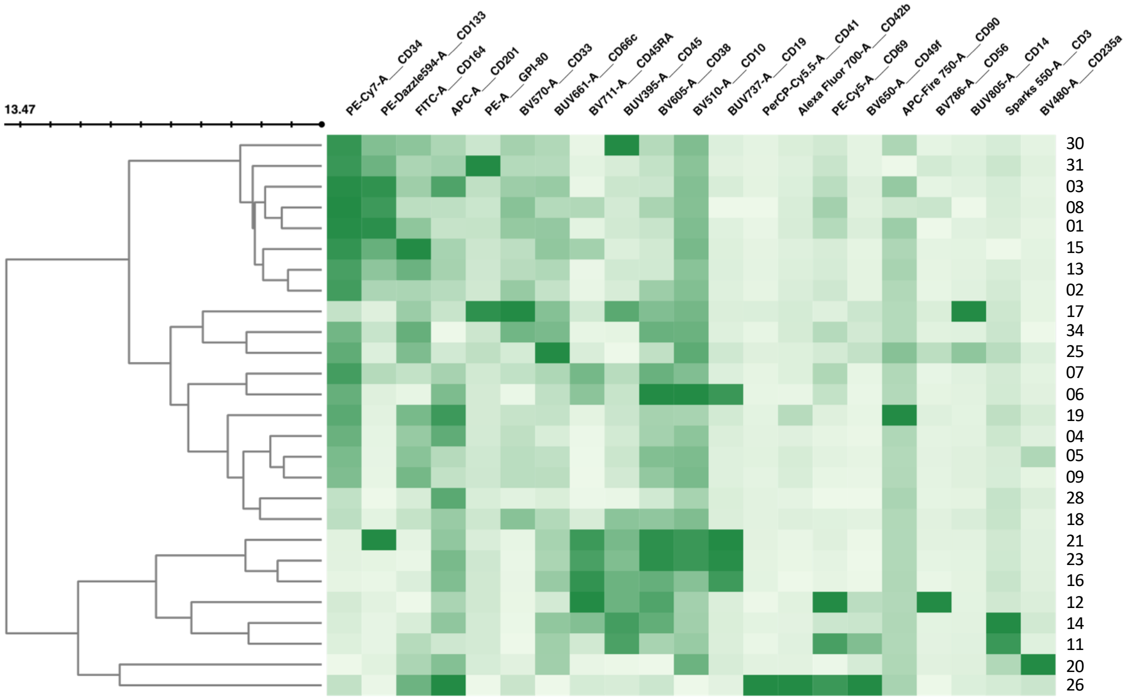

c

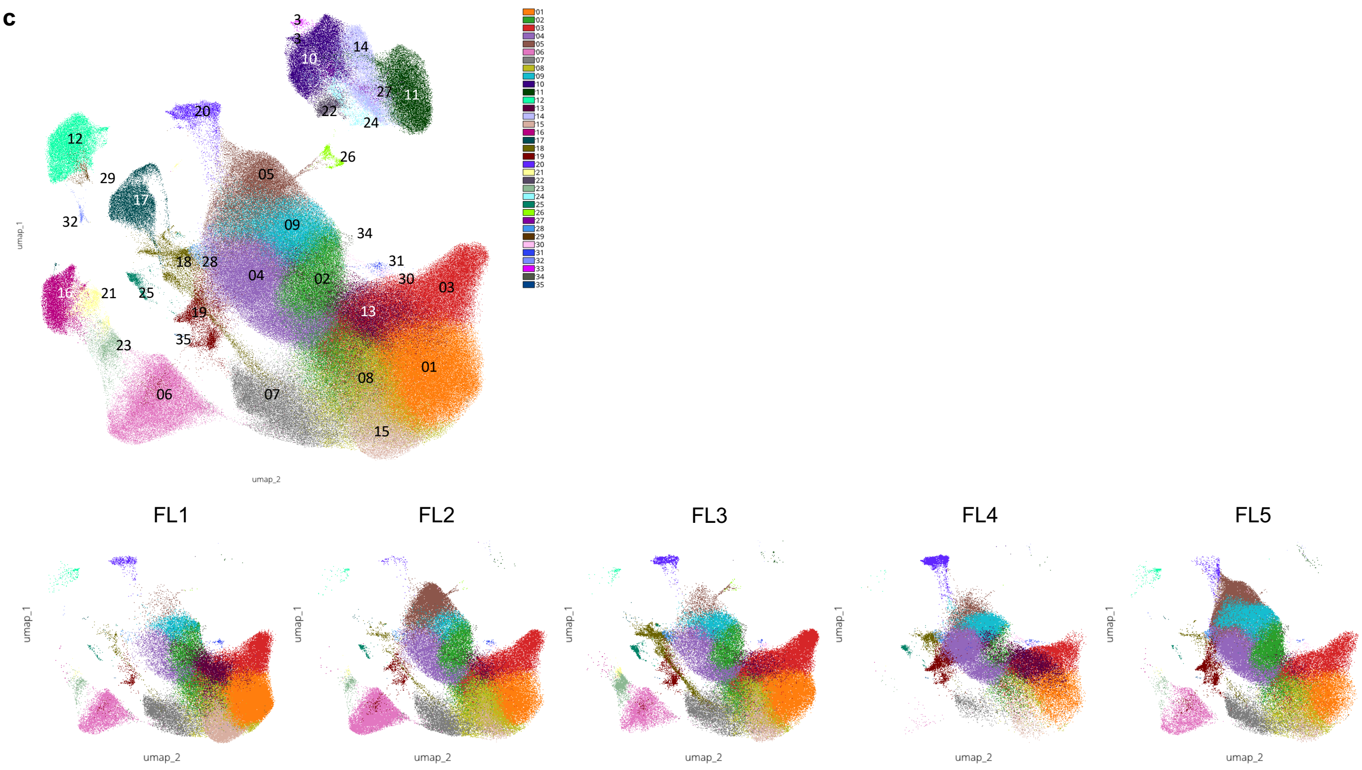

Figure S6

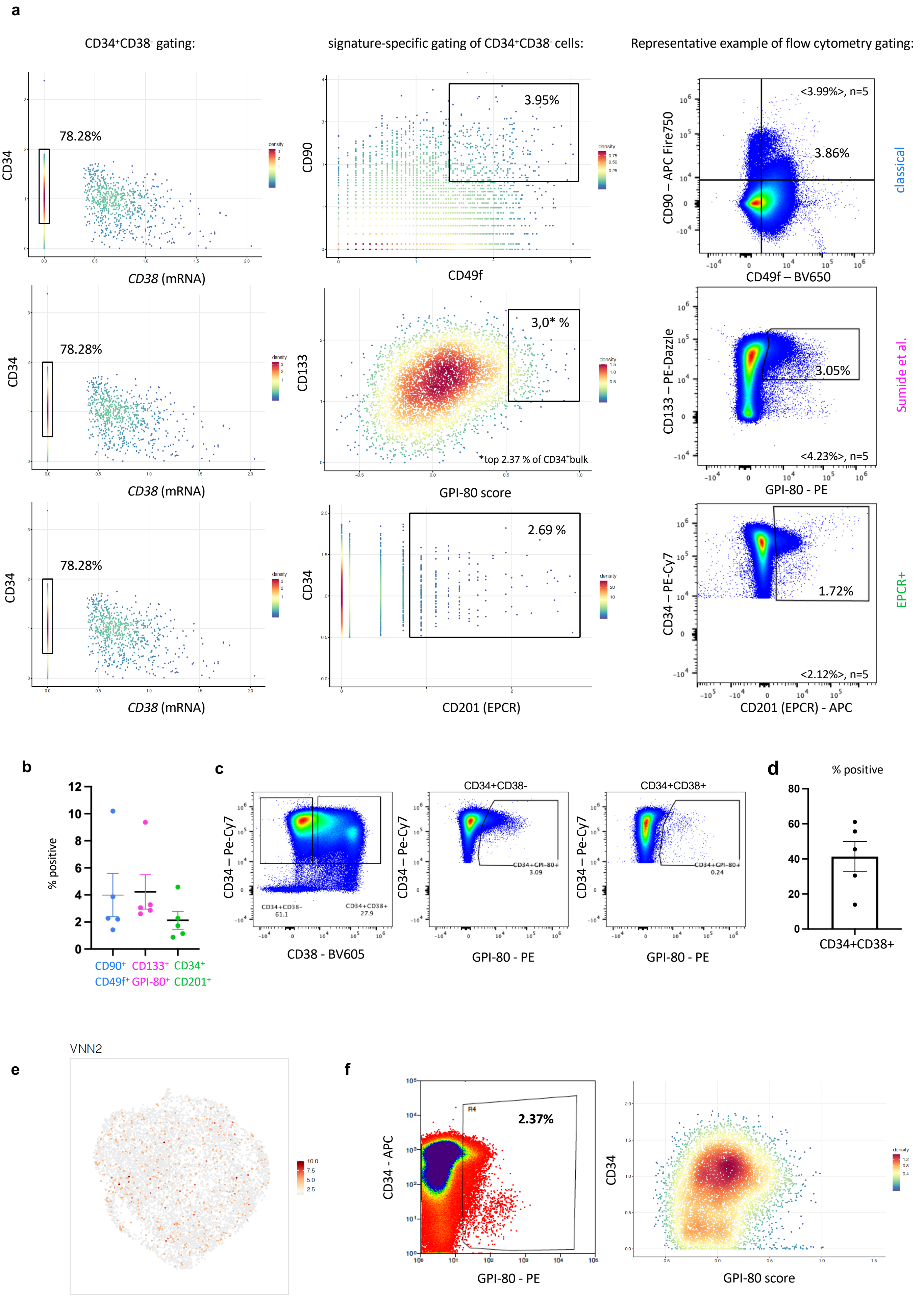
a
CD34+CD38- gating:
signature-specific gating of CD34+CD38- cells:
Representative example of flow cytometry gating:
78.28%
CD34
CD38 (mRNA)
3.95%
CD90
CD49f
<3.99%>, n=5
3.86%
CD90 – APC Fire750
CD49f – BV650
classical
3,0* %
*top 2.37 % of CD34+bulk
CD133
GPI-80 score
CD133 – PE-Dazzle
3.05%
<4.23%>, n=5
GPI-80 - PE
Sumide et al.
2.69 %
CD201 (EPCR)
CD34 – PE-Cy7
1.72%
<2.12%>, n=5
CD201 (EPCR) - APC
EPCR+
b
% positive
CD90+ CD49f+
CD133+ GPI-80+
CD34+ CD201+
c
CD34 – Pe-Cy7
CD38 - BV605
CD34+CD38- 61.1
CD34+CD38+ 27.9
CD34+CD38-
CD34+GPI-80+ 3.09
CD34+CD38+
CD34+GPI-80+ 0.24
GPI-80 - PE
d
% positive
CD34+CD38+
e
VNN2
f
CD34 - APC
R4
2.37%
GPI-80 - PE
CD34
GPI-80 score

Figure S7

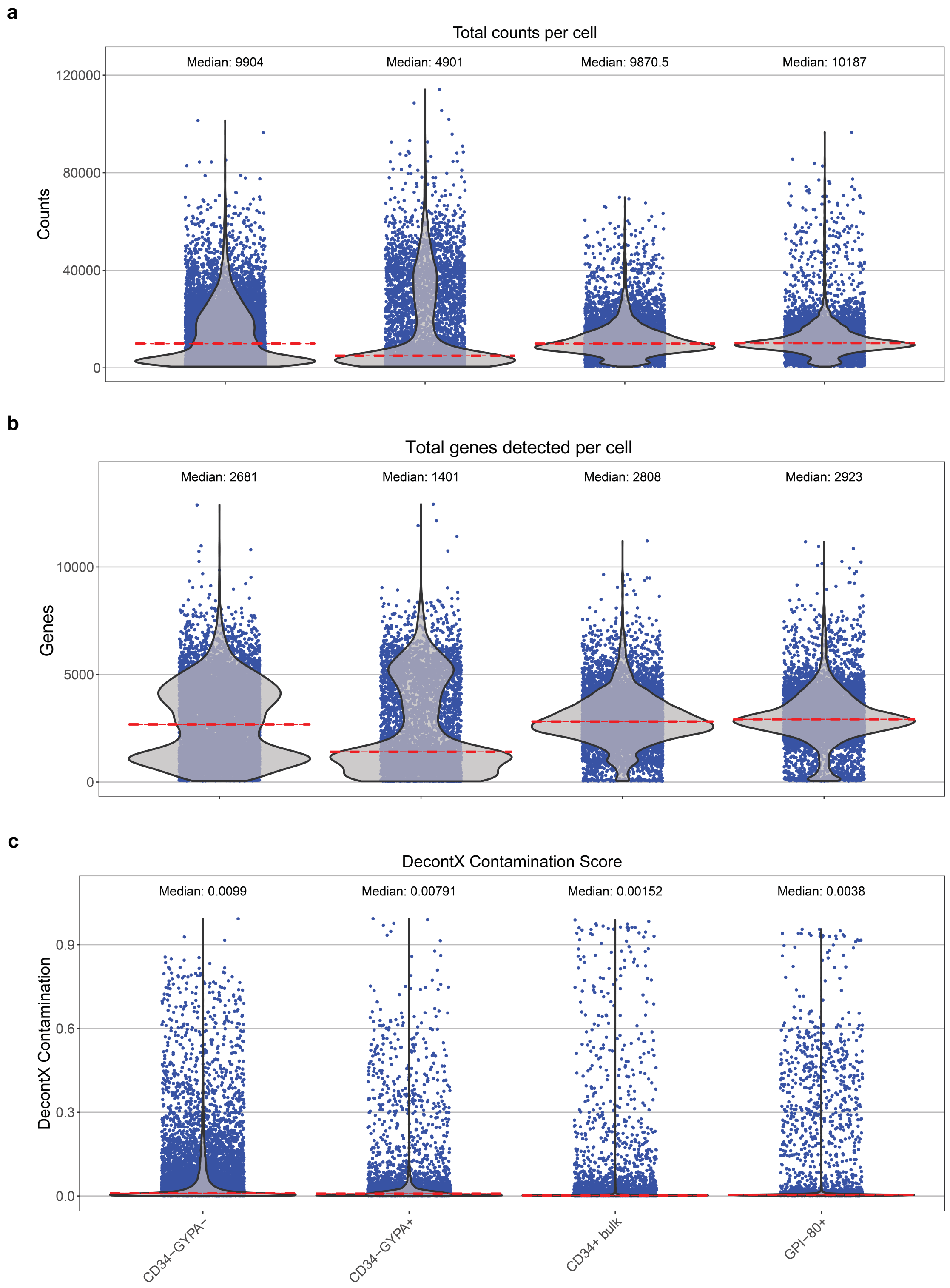

a
Total counts per cell
Median: 9904
Median: 4901
Median: 9870.5
Median: 10187
Counts
120000
80000
40000
0
b
Total genes detected per cell
Median: 2681
Median: 1401
Median: 2808
Median: 2923
Genes
10000
5000
0
c
DecontX Contamination Score
Median: 0.0099
Median: 0.00791
Median: 0.00152
Median: 0.0038
DecontX Contamination
0.9
0.6
0.3
0.0
CD34−GYPA−
CD34−GYPA+
CD34+ bulk
GPI−80+

SUPPLEMENTARY TABLE LEGENDS

Supplementary Table 1. Oligo-tagged antibodies used in CITE-Seq

The antibodies included in this panel consist of known markers relevant to characterize HSCs and other candidates identified in a pilot experiment.

Supplementary Table 2. Differentially expressed genes (DEGs) between GPI-80^{+} and CD34^{+} bulk HSCs/MPPs

P-values were generated by MAST (wrapped in Seurat), which uses a two-sided likelihood ratio test. P-values were adjusted for multiple comparisons using a bonferroni correction based on the total number of genes in the dataset.

Supplementary Table 3. Differentially expressed genes (DEGs) between clusters within the GPI-80^{+} fraction

P-values were generated by MAST (wrapped in Seurat), which uses a two-sided likelihood ratio test. P-values were adjusted for multiple comparisons using a bonferroni correction based on the total number of genes in the dataset.

Supplementary Table 4. Top enriched genes per cluster in the different HSC/ MPP fractions in Figures 2c-e

The rank is indicated behind the genes and the bolded gene names were selected for labeling of the different clusters in **Figure 2e**.

Supplementary Table 5. Multi-dimensional flow cytometry panel antibodies

Supplementary Table 1. Oligo-tagged antibodies used in CITE-Seq

Cell surface marker	Gene	Vendor	Cat #	Concentration
CD34	*CD34*	BioLegend	343537	1ug in 100ul
CD38	*CD38*	BioLegend	303541	1ug in 100ul
CD90	*THY1*	BioLegend	328135	1ug in 100ul
CD45	*PTPRC*	BioLegend	304064	1ug in 100ul
CD49f	*ITGA6*	BioLegend	313633	1ug in 100ul
CD133	*PROM1*	BioLegend	372815	1ug in 100ul
CD201	*EPCR/PROCR*	BioLegend	351907	1ug in 100ul
CD164	*CD164*	BioLegend	324809	1ug in 100ul
CD105	*ENG*	BioLegend	323221	1ug in 100ul
CD144	*CDH5/ VECAD*	BioLegend	348517	1ug in 100ul
CD202b	*TEK/TIE2*	BioLegend	334213	1ug in 100ul
VEGFR3	*FLT4*	BioLegend	356207	1ug in 100ul
CLEC1B	*CLEC1B*	BioLegend	372009	1ug in 100ul
CD31	*PECAM1*	BioLegend	303137	1ug in 100ul
CD184	*CXCR4*	BioLegend	306531	1ug in 100ul
HLA-DR,DP,DQ	*HLA class II genes*	BioLegend	307659	1ug in 100ul
pan-HLA class I	*HLA class I genes*	BioLegend	311445	1ug in 100ul
CD141	*THBD*	BioLegend	344121	1ug in 100ul
CD235a	*GYPA*	BioLegend	349117	1ug in 100ul

Supplementary Table 4. Top enriched genes in HSC/MPP clusters across samples

cluster	HSC/MPP (Figure 2C)	CD34+ bulk (Figure 2D)	GPI-80+ (Figure 2E)
HSC/MPP 0	ID1 (1), VIM (2), RGCC (3), **LMNA (4)**	RGCC (1), **LMNA (2)**	RGCC (1), **LMNA (2)**
HSC/MPP 1	**CDK6 (1)**	IGLL1 (1), HCST (2), **CDK6 (3)**	IGLL1 (1), HCST (2), **CDK6 (3)**
HSC/MPP 2	**FOS (1), JUN (2)**	**FOS (1), JUN (2)**	**FOS (1), JUN (2)**
HSC/MPP 3	HSPA1A (1), **HSPA1B (2)**	HSPA1A (1), **HSPA1B (2)**	**HSPA1B (1)**, HSPA1A (2)
HSC/MPP 4	**HIST1H4C (1)**	**HIST1H4C (1)**	**HIST1H4C (1)**
pDC/ DC pre	**IGLL1 (1**), LTB (2), IGJ (3)	**IGLL1 (1)**, LTB (2), IGJ (3)	**IGLL1 (1)**, LTB (2), IGJ (3)
neutrophil/myeloid progenitor	**MPO (1)**, PRTN3 (2), AZU1 (3), LFD (4), IGLL1 (5)	IGLL1 (1), **MPO (2)**	IGLL1 (1), **MPO (2)**
HSC/MPP 5	**PRSS3P1 (1)**	**PRSS3P1 (1)**	**PRSS3P1 (1)**
HSC/MPP 6	**CENPF (1)**, TOP2A (2)	**CENPF (1)**, TOP2A (2)	**CENPF (1)**, TOP2A (2)

Supplementary Table 5. Multi-dimensional flow cytometry panel antibodies

marker	Fluorophore	Vendor	Cat #	Dilution
CD45	BUV395	BD Biosciences	563791	1:100
Live/dead	fixable UV live/dead	Thermo Fischer	L34961	1:800
CD66c	BUV661	BD Biosciences	741653	1:50
CD19	BUV737	BD Biosciences	612757	1:100
CD14	BUV805	BD Biosciences	612902	1:50
CD235a	Pacific Blue	BD Biosciences	746358	1:400
CD10	BV510	BioLegend	312219	1:50
CD33	BV570	BioLegend	303417	1:400
CD38	BV605	BioLegend	303532	1:50
CD49f	BV650	BD Biosciences	563706	1:200
CD45RA	BV711	BioLegend	304137	1:100
CD56	BV786	BD Biosciences	564058	1:100
CD164	FITC	BioLegend	324805	1:50
CD3	Spark Blue 550	BioLegend	100259	1:100
GPI-80	PE	MBL International	D087-5	1:50
CD133	PE-Dazzle 594	BioLegend	372811	1:100
CD41	PerCP-Cy55	BioLegend	303719	1:100
CD69	PE-Cy5	BioLegend	310907	1:25
CD34	PE-Cy7	BioLegend	343515	1:100
CD201 (EPCR)	APC	BioLegend	351906	1:25
CD42b	Alexa 700	BioLegend	303927	1:25
CD90	APC-Fire750	BioLegend	328137	1:25

SUPPLEMENTARY REFERENCES

1. Monaco, G. *et al.* RNA-Seq Signatures Normalized by mRNA Abundance Allow Absolute Deconvolution of Human Immune Cell Types. *Cell Rep* **26**, 1627-1640 e1627 (2019).
2. Zheng, S., Papalexi, E., Butler, A., Stephenson, W. & Satija, R. Molecular transitions in early progenitors during human cord blood hematopoiesis. *Mol Syst Biol* **14**, e8041 (2018).
3. Belluschi, S. *et al.* Myelo-lymphoid lineage restriction occurs in the human haematopoietic stem cell compartment before lymphoid-primed multipotent progenitors. *Nat Commun* **9**, 4100 (2018).
4. Velten, L. *et al.* Human haematopoietic stem cell lineage commitment is a continuous process. *Nat Cell Biol* **19**, 271-281 (2017).
