# Supplementary material for: Multi-modal profiling of human fetal liver hematopoietic stem cells reveals the molecular signature of engraftment
Source: Nat Commun. 2022 Mar 1;13:1103. doi: 10.1038/s41467-022-28616-x (PMC8888592; doi:10.1038/s41467-022-28616-x)
Supplement: Supplementary file 6 — Reporting Summary (previous version) [file 41467_2022_28616_MOESM6_ESM.pdf]

nature research

Corresponding author(s): Kim Vanuytsel, George J Murphy, Alejandro B Balazs

Last updated by author(s): Dec 24, 2021

Reporting Summary

Nature Research wishes to improve the reproducibility of the work that we publish. This form provides structure for consistency and transparency in reporting. For further information on Nature Research policies, see our Editorial Policies and the Editorial Policy Checklist.

Statistics

For all statistical analyses, confirm that the following items are present in the figure legend, table legend, main text, or Methods section.

n/a	Confirmed	
☐	☒	The exact sample size (*n*) for each experimental group/condition, given as a discrete number and unit of measurement
☐	☒	A statement on whether measurements were taken from distinct samples or whether the same sample was measured repeatedly
☐	☒	The statistical test(s) used AND whether they are one- or two-sided<br>*Only common tests should be described solely by name; describe more complex techniques in the Methods section.*
☒	☐	A description of all covariates tested
☒	☐	A description of any assumptions or corrections, such as tests of normality and adjustment for multiple comparisons
☐	☒	A full description of the statistical parameters including central tendency (e.g. means) or other basic estimates (e.g. regression coefficient) AND variation (e.g. standard deviation) or associated estimates of uncertainty (e.g. confidence intervals)
☐	☒	For null hypothesis testing, the test statistic (e.g. *F*, *t*, *r*) with confidence intervals, effect sizes, degrees of freedom and *P* value noted<br>*Give P values as exact values whenever suitable.*
☒	☐	For Bayesian analysis, information on the choice of priors and Markov chain Monte Carlo settings
☒	☐	For hierarchical and complex designs, identification of the appropriate level for tests and full reporting of outcomes
☒	☐	Estimates of effect sizes (e.g. Cohen's *d*, Pearson's *r*), indicating how they were calculated

Our web collection on statistics for biologists contains articles on many of the points above.

Software and code

Policy information about availability of computer code

Data collection	Spectroflo v2.2.1 on the Aurora (Cytek Biosciences), CellQuest v.4.1 on the Stratedigm S1300 (Stratedigm), Summit v6.3.1 on the MoFlo Astrios (Beckman Coulter)
Data analysis	Statistical analysis was performed using Prism (versions 9.1.2 and 9.2.0), flow cytometry data analysis was performed in FlowJo (versions 10.2.0, 10.7.0 - 10.8.0) and data analysis pipelines for the multi-parameter flow cytometry experiments were built using cloud-based OMIQ analysis platform (Omiq.ai), Computational analysis of the sequencing data was performed using Seurat v.3.2.1, cell ranger v.3.0.2, CITE-Seq-Count v 1.4.2, bcl2fastq v.2.2, singleCellTK package v.1.99.1, DecontX (in celda v.1.9.1), MAST v.1.2.1 (used as wrapper with Seurat), Monocle v.3.2, SCENIC<br><br>Code availability:<br>The code used in this work is available at: https://github.com/CReM-BU/Vanuytsel_hPSC<br>Multiparameter flow cytometry code (unmixed fluorescence cytometry dataset with integrated UMAP and Phenograph parameters) is available at: http://flowrepository.org/id/FR-FCM-Z32M

For manuscripts utilizing custom algorithms or software that are central to the research but not yet described in published literature, software must be made available to editors and reviewers. We strongly encourage code deposition in a community repository (e.g. GitHub). See the Nature Research guidelines for submitting code & software for further information.

Data

Policy information about availability of data

All manuscripts must include a data availability statement. This statement should provide the following information, where applicable:

- Accession codes, unique identifiers, or web links for publicly available datasets
- A list of figures that have associated raw data
- A description of any restrictions on data availability

The raw CITE-seq data generated in this study has been deposited in the GEO database under accession number GSE160251:
https://www.ncbi.nlm.nih.gov/geo/query/acc.cgi?acc=GSE160251

The CITE-seq data generated in this study is also available through the following interactive browser:
https://engraftable-hsc.cells.ucsc.edu

The multi-parameter flow cytometry data generated in this study (unmixed fluorescence cytometry dataset) has been deposited in the FlowRepository database:
http://flowrepository.org/id/FR-FCM-Z32M

The human genome (GRCh38) sequence data used in this study is available through the Ensembl genome browser: http://ftp.ensembl.org/pub/release-104/fasta/homo_sapiens/dna/
The mouse genome (GRCm38) sequence data used in this study is available through the Ensembl genome browser: http://ftp.ensembl.org/pub/release-104/fasta/mus_musculus/dna/

Source data are provided with this paper.

Field-specific reporting

Please select the one below that is the best fit for your research. If you are not sure, read the appropriate sections before making your selection.

- [x] Life sciences
- [ ] Behavioural & social sciences
- [ ] Ecological, evolutionary & environmental sciences

For a reference copy of the document with all sections, see nature.com/documents/nr-reporting-summary-flat.pdf

Life sciences study design

All studies must disclose on these points even when the disclosure is negative.

Sample size	No sample size calculation was performed. CITE-seq analysis was performed on one fetal liver sample as the cost was prohibitive to profile several biological repeats at this resolution. The observed expression patterns were validated across 5 additional fetal liver samples using an orthogonal approach (multi-parameter cytometry).
Data exclusions	During CITE-seq analysis and quality control, cells with more than 25% of reads mapping to mitochondrial genes were filtered out. A cluster representing cells with high mitochondrial content and mixed lineage identities was also excluded from downstream analysis. This is indicated in the methods. The exclusion criteria were not pre-established and reflect what is commonly done in the field in terms of quality control.
Replication	Multi-dimensional flow cytometry characterization confirmed the expression patterns observed based on antibody-derived tag sequencing in the CITE-seq experiment and validated these findings across five biologically distinct fetal liver samples
Randomization	N/A: There were no different experimental groups
Blinding	N/A: There were no different experimental groups

Reporting for specific materials, systems and methods

We require information from authors about some types of materials, experimental systems and methods used in many studies. Here, indicate whether each material, system or method listed is relevant to your study. If you are not sure if a list item applies to your research, read the appropriate section before selecting a response.

Materials & experimental systems

n/a	Involved in the study
☐	☒ Antibodies
☐	☒ Eukaryotic cell lines
☒	☐ Palaeontology and archaeology
☐	☒ Animals and other organisms
☒	☐ Human research participants
☒	☐ Clinical data
☒	☐ Dual use research of concern

Methods

n/a	Involved in the study
☒	☐ ChIP-seq
☐	☒ Flow cytometry
☒	☐ MRI-based neuroimaging

Antibodies

Antibodies used

A list of the antibodies used is provided below. These are all commercially available antibodies and have been validated by the manufacturer for their target and application. Validation information, recommended dilution and references are all available on the manufacturer's website and can be accessed through the links provided in this antibody list as well as by using the catalog numbers listed in Supplementary tables 1 and 5.

CITE-Seq antibodies:
CD34 (clone 561) 343537 BioLegend https://www.biolegend.com/en-ie/products/totalseq-a0054-anti-human-cd34-antibody-16197
CD38 (clone HB-7) 303541 BioLegend https://www.biolegend.com/en-ie/products/totalseq-a0389-anti-human-cd38-antibody-16899
CD90 (clone 5E10) 328135 BioLegend https://www.biolegend.com/en-ie/products/totalseq-a0060-anti-human-cd90-thy1-antibody-15928
CD45 (clone HI30) 304064 BioLegend https://www.biolegend.com/en-ie/products/totalseq-a0391-anti-human-cd45-antibody-15934
CD49f (clone GoH3) 313633 BioLegend https://www.biolegend.com/en-ie/products/totalseq-a0070-anti-human-mouse-cd49f-antibody-15953
CD133 (clone 7) 372815 BioLegend https://www.biolegend.com/en-ie/products/totalseq-a0126-anti-human-antibody-cd133-16393
CD201 (clone RCR-401) 351907 BioLegend https://www.biolegend.com/en-ie/products/0069-anti-human-cd201-epcr-antbody-16227
CD164 (clone 67D2) 324809 BioLegend https://www.biolegend.com/en-ie/products/totalseq-a0821-anti-human-cd164-antibody-17204
CD105 (clone 43A3) 323221 BioLegend https://www.biolegend.com/en-ie/products/totalseq-a0068-anti-human-cd105-antibody-16346
CD144 (clone BV9) 348517 BioLegend https://www.biolegend.com/en-ie/products/totalseq-a0400-anti-human-cd144-ve-cadherin-antibody-16789
CD202b (clone 33.1 (Ab33)) 334213 BioLegend https://www.biolegend.com/en-ie/products/totalseq-a0588-anti-human-cd202b-tie2-tek-antibody-17853
VEGFR3 (clone 9D9F9) 356207 BioLegend https://www.biolegend.com/en-ie/products/totalseq-a0865-anti-human-vegfr-3-flt-4-antibody-18846
CLEC1B (clone AYPI) 372009 BioLegend https://www.biolegend.com/en-ie/products/totalseq-a0866-anti-human-clec1b-clec2-antibody-18098
CD31 (clone WM59) 303137 BioLegend https://www.biolegend.com/en-ie/products/totalseq-a0124-anti-human-cd31-antibody-16332
CD184 (clone 12G5) 306531 BioLegend https://www.biolegend.com/en-ie/products/totalseq-a0366-anti-human-cd184-cxcr4-antibody-17277
HLA-DR,DP,DQ (clone L243) 307659 BioLegend https://www.biolegend.com/en-ie/products/totalseq-a0159-anti-human-hla-dr-antibody-16189
pan-HLA class I (clone W6/32) 311445 BioLegend https://www.biolegend.com/en-ie/products/totalseq-a0058-anti-human-hla-a-b-c-antibody-16175
CD141 (clone M80) 344121 BioLegend https://www.biolegend.com/en-ie/products/totalseq-a0163-anti-human-cd141-thrombomodulin-antibody-16639
CD235a (clone HI264) 349117 BioLegend https://www.biolegend.com/en-ie/products/totalseq-a0574-anti-human-cd235a-glycophorin-a-antibody-16774

multi-parameter flow cytometry panel antibodies:
CD45 BUV395 (clone HI30) 563791 BD Biosciences https://www.bdbiosciences.com/us/applications/research/stem-cell-research/cancer-research/human/buv395-mouse-anti-human-cd45-hi30/p/563791
livedead fixable UV livedead L34961 Thermo Fischer https://www.thermofisher.com/order/catalog/product/L34961?SID=srch-srp-L34961#/L34961?SID=srch-srp-L34961
CD66c BUV661 (clone B1.1/CD66) 741653 BD Biosciences https://www.bdbiosciences.com/us/reagents/research/antibodies-buffers/buv661-mouse-anti-human-cd66-b11cd66/p/741653
CD19 BUV737 (clone SJ25C1) 612757 BD Biosciences https://www.bdbiosciences.com/us/reagents/research/antibodies-buffers/immunology-reagents/anti-human-antibodies/cell-surface-antigens/buv737-mouse-anti-human-cd19-sj25c1-also-known-as-sj25-c1/p/612757
CD14 BUV805 (clone M5E2) 612902 BD Biosciences https://www.bdbiosciences.com/us/reagents/research/antibodies-buffers/immunology-reagents/anti-human-antibodies/cell-surface-antigens/buv805-mouse-anti-human-cd14-m5e2/p/612902
CD235a BV480 (clone GA-R2 (HIR2)) 746358 BD Biosciences https://www.bdbiosciences.com/us/reagents/research/antibodies-buffers/immunology-reagents/anti-human-antibodies/cell-surface-antigens/bv480-mouse-anti-human-cd235a-ga-r2-hir2/p/746358?cc=US

CD10 BV510 (clone HI10a) 312219 BioLegend https://www.biolegend.com/en-us/products/brilliant-violet-510-anti-human-cd10-antibody-8306
CD33 BV570 (clone WM53) 303417 BioLegend https://www.biolegend.com/en-us/products/brilliant-violet-570-anti-human-cd33-antibody-7468
CD38 BV605 (clone HIT2) 303532 BioLegend https://www.biolegend.com/en-us/products/brilliant-violet-605-anti-human-cd38-antibody-8154
CD49f BV650 (clone GoH3) 563706 BD Biosciences https://www.bdbiosciences.com/us/applications/research/stem-cell-research/cancer-research/human/bv650-rat-anti-human-cd49f-goh3/p/563707
CD45RA BV711 (clone HI100) 304137 BioLegend https://www.biolegend.com/en-us/products/brilliant-violet-711-anti-human-cd45ra-antibody-7937
CD56 BV786 (clone NCAM16.2) 564058 BD Biosciences https://www.bdbiosciences.com/us/reagents/research/antibodies-buffers/immunology-reagents/anti-human-antibodies/cell-surface-antigens/bv786-mouse-anti-human-cd56-ncam162-also-known-as-ncam-16/p/564058
CD164 FITC (clone 67D2) 324805 BioLegend https://www.biolegend.com/en-us/search-results/fitc-anti-human-cd164-antibody-3805
CD3 Spark Blue 550 (clone 17A2) 100259 BioLegend https://www.biolegend.com/en-us/search-results?Clone=17A2
GPI-80 PE (clone 3H9) D087-5 MBL https://www.mblintl.com/products/d087-5/
CD133 PE-Dazzle 594 (clone 7) 372811 Biolegend https://www.biolegend.com/en-us/search-results/pe-dazzle-594-anti-human-cd133-antibody-14207
CD41 PerCP-Cy55 (clone HIP8) 303719 BioLegend https://www.biolegend.com/en-us/products/percp-cyanine5-5-anti-human-cd41-antibody-8314
CD69 PE-Cy5 (clone FN50) 310907 BioLegend https://www.biolegend.com/en-us/search-results/pe-cyanine5-anti-human-cd69-antibody-1673
CD34 PE-Cy7 (clone 581) 343515 BioLegend https://www.biolegend.com/en-us/products/pe-cy7-anti-human-cd34-antibody-6160
CD201 (EPCR) APC (clone RCR-401) 351906 Biolegend https://www.biolegend.com/en-us/products/apc-anti-human-cd201-epcr-antibody-7693
CD42b Alexa 700 (clone HIP1) 303927 Biolegend https://www.biolegend.com/en-us/products/alexa-fluor-700-anti-human-cd42b-antibody-14765
CD90 APC-Fire750 (clone 5E10) 328137 Biolegend https://www.biolegend.com/en-us/search-results/apc-fire-750-anti-human-cd90-thy1-antibody-15884

Antibodies used for transplantation read-outs:
hCD4 BV421 (clone RPA-T8) 300532 BioLegend https://www.biolegend.com/en-us/products/brilliant-violet-421-anti-human-cd4-antibody-7151
hCD3 BV605 (clone UCHT1) 300460 BioLegend https://www.biolegend.com/en-us/products/brilliant-violet-605-anti-human-cd3-antibody-10421
hCD20 BV650 (clone 2H7) 302336 BioLegend https://www.biolegend.com/en-us/products/brilliant-violet-650-anti-human-cd20-antibody-7876
hCD45 BV785 (clone HI30) 304048 BioLegend https://www.biolegend.com/en-us/products/brilliant-violet-785-anti-human-cd45-antibody-9325
hCD33 PE (clone WM53) 303404 BioLegend https://www.biolegend.com/en-us/products/pe-anti-human-cd33-antibody-878
mCD45 PE-Dazzle594 (clone 30-F11)
103146 BioLegend https://www.biolegend.com/en-us/products/pe-dazzle-594-anti-mouse-cd45-antibody-10070
hCD14 PerCP-Cy5.5 (clone HCD14) 325622 BioLegend https://www.biolegend.com/en-us/products/pe-dazzle-594-anti-mouse-cd45-antibody-10070
hCD8 APC (RPA-T8) 301049 BioLegend https://www.biolegend.com/en-us/products/apc-anti-human-cd8a-antibody-831
hCD45 APC (clone HI30)
304012 BioLegend https://www.biolegend.com/en-us/products/apc-anti-human-cd45-antibody-705
mCD45 PE-Dazzle594 (clone 30-F11) 103126 BioLegend https://www.biolegend.com/en-us/products/pacific-blue-anti-mouse-cd45-antibody-3102

Validation

The antibodies used in this work are all commercially available antibodies and have been validated by the manufacturer for their target and application. Validation information, recommended dilution and references are all available on the manufacturer's website and can be accessed through the links provided in this antibody list as well as by using the catalog numbers listed in Supplementary tables 1 and 5. All antibodies used in the multi-parameter flow cytometry experiment were specifically titrated during panel design for optimal resolution.

Eukaryotic cell lines

Policy information about cell lines

Cell line source(s)

mouse embryonic stem cells (mESCs) from Nkx2-1mCherry mice were spiked into samples prepped for CITE-Seq to control for background staining from the TotalSeq A antibodies.

Authentication

Describe the authentication procedures for each cell line used OR declare that none of the cell lines used were authenticated.

Mycoplasma contamination

All cell lines used tested negative for mycoplasma.

Commonly misidentified lines
(See ICLAC register)

Name any commonly misidentified cell lines used in the study and provide a rationale for their use.

Animals and other organisms

Policy information about studies involving animals; ARRIVE guidelines recommended for reporting animal research

Laboratory animals	13-16 week old female NOD/SCID/IL2rynull (NSG) mice (JAX NSG 005557 - NOD.Cg-Prkdc<scid> Il2rg<tm1Wjl>/SzJ) were used for transplantation experiments. The mice were housed under the following conditions: temperature 68-73F, humidity 30-70%, light cycle 12/12: 7AM-7PM light, 7PM-7AM dark
Wild animals	The study did not involve wild animals.
Field-collected samples	The study did not involve samples collected from the field.
Ethics oversight	All mouse research complied with the Institutional Animal Care and Use Committee (IACUC) of the Massachusetts General Hospital (Protocol #2009N000136)

Note that full information on the approval of the study protocol must also be provided in the manuscript.

Flow Cytometry

Plots

Confirm that:

- [x] The axis labels state the marker and fluorochrome used (e.g. CD4-FITC).
- [x] The axis scales are clearly visible. Include numbers along axes only for bottom left plot of group (a 'group' is an analysis of identical markers).
- [x] All plots are contour plots with outliers or pseudocolor plots.
- [x] A numerical value for number of cells or percentage (with statistics) is provided.

Methodology

Sample preparation

This study was reviewed by the Mass General Brigham Institutional Review Board (IRB Protocol #2016P001106) and was determined not to constitute human subjects research given its use of de-identified, discarded material. All human donor tissues were obtained under the approved protocol from participants who consented in writing to the use of donations for research. Participants received no compensation.

Processing of fetal liver (FL) samples:
The FL sample used for CITE-seq was collected at 21 post conception weeks (pcw) (sex NA). For the multiparameter flow characterization the following 5 samples were used: FL1 (21 pcw, sex N/A), FL2 (22 pcw, F), FL3 (16 pcw, sex N/A), FL4 (17 pcw, sex N/A), FL5 (17 pcw, F). FL samples were mechanically dissociated into small pieces and incubated in Liver Digest Medium (Fisher Scientific, 17703034) at 37C. Mononuclear cells were isolated over a Ficoll gradient (Lymphoprep: Stem Cell Technologies, 7851) prior to separation into CD34+ and flowthrough (CD34-) cells using magnetic beads (CD34 Microbead Kit: Miltenyi Biotec, 130-046-702).
FL cells were thawed and allowed to recover at 37C for an hour prior to staining. Cells were blocked with TruStain FcX (BioLegend, 422301) and stained with TotalSeq A antibody mix containing 1ug of each TotalSeq A antibody per condition (BioLegend, see Table S1 for a list of antibodies). Anti-human CD235a-APC antibody (BD Biosciences, 551336) was added to the CD34- fraction. The CD34+ fraction was stained with anti-human CD34-APC antibody (BD Biosciences, 555824) and anti-human GPI-80-PE antibody (MBL International, D087-5). All samples were stained with calcein blue (Invitrogen, C34853) for live/dead exclusion prior to sorting.

Processing of peripheral blood mononuclear cells (PBMCs):
PBMCs from a 65-year-old individual (M) were used as a control for the flow cytometry experiments. Mononuclear cells were isolated from a Leukoreduction System (LRS) Chamber (New York Biologics Inc, M, age 65) over a Ficoll gradient (Cytiva, 17144003).

For the multi-parameter flow cytometry analysis, PBMC and FL cells were thawed, washed, blocked with Human FcBlock (BioLegend, 422301) and stained with Live Dead Blue amine dye (Thermo Fisher, L34961). A cocktail of antibodies (Supplementary table 5) was prepared fresh and supplemented with Monocyte Blocker (BioLegend, 426102) and Brilliant Buffer Plus (BD Biosciences, 566385). Cells were stained in the dark at 4C and washed twice. A sub-panel of brightly-expressed markers was assessed as a separate stain to serve as a fluorescence-minus-multiple control for further data interpretation. PBMC cells and single stain Ultracomp beads (Thermo Fisher, 01-2222-42) were stained to provide single stain spectral controls.

Instrument

Cells and single stain controls were analyzed on a 5-laser Aurora spectral flow cytometry (Cytek Biosciences) and raw fluorescence data from 64 channels were unmixed using ordinary least square algorithm in Spectraflo v2.2.1 (Cytek Biosciences). For cell sorting, a Beckman Coulter MoFlo Astrios device was used with Summit v6.3.1 software. Transplantation experiments were read out on a Stratedign S1300 with CellQuest v.4.1 software.

Software	Flowjo was used to analyze flow cytometry data. For the multi-parameter flow experiment, data analysis pipelines were built using cloud-based OMIQ analysis platform (Omiq). Detailed information is available in the methods section.
Cell population abundance	This information is included in the figure legends where appropriate (e.g Supplementary Figure 1a)
Gating strategy	For the sorting experiments, initial gating steps consist of gating out cell debris based on SSC/FSC parameters and subsequent doublet exclusion. Calcein blue staining was used to gate out dead (calcein blue-) cells. Further gating for particular markers was based on comparison to a sample not stained with those markers. The final sort gates are depicted in Figure S1 for all fractions.<br><br>Similarly, for all other flow cytometry experiments, initial gating steps consist of gating out cell debris based on SSC/FSC. Figure S6a shows representative flow plots (right) to guide the in silico sorting (left) and to match the in silico gated fractions, CD34+CD38- cells were selected prior to further signature-specific gating. The latter is illustrated in Figure S6a. A representative example of the prior CD34+CD38- gating step is also presented in figure S6c with the relevant gates shown. GPI-80+ gating was realized by comparing to cells that did not receive GPI-80 antibody staining. CD201+ gating was realized by comparing to cells that did not receive CD201 antibody staining. CD90+CD49f+ gating was realized by comparing to cells that were not stained by either antibody.

[x] Tick this box to confirm that a figure exemplifying the gating strategy is provided in the Supplementary Information.
